# Precision Breast Cancer Medicine: Early Stage Triple Negative Breast Cancer—A Review of Molecular Characterisation, Therapeutic Targets and Future Trends

**DOI:** 10.3389/fonc.2022.866889

**Published:** 2022-08-08

**Authors:** Karen Pinilla, Lynsey M. Drewett, Rebecca Lucey, Jean E. Abraham

**Affiliations:** ^1^ Precision Breast Cancer Institute, University of Cambridge, Cambridge, United Kingdom; ^2^ Department of Oncology, Cambridge University Hospitals NHS Foundation Trust, Cambridge, United Kingdom; ^3^ Cancer Research UK Cambridge Centre, Cancer Research UK Cambridge Institute, University of Cambridge, Cambridge, United Kingdom

**Keywords:** Breast cancer, precision medicine, triple negative, therapeutic, target, biomarkers, early disease

## Abstract

Personalised approaches to the management of all solid tumours are increasing rapidly, along with wider accessibility for clinicians. Advances in tumour characterisation and targeted therapies have placed triple-negative breast cancers (TNBC) at the forefront of this approach. TNBC is a highly heterogeneous disease with various histopathological features and is driven by distinct molecular alterations. The ability to tailor individualised and effective treatments for each patient is of particular importance in this group due to the high risk of distant recurrence and death. The mainstay of treatment across all subtypes of TNBC has historically been cytotoxic chemotherapy, which is often associated with off-target tissue toxicity and drug resistance. Neoadjuvant chemotherapy is commonly used as it allows close monitoring of early treatment response and provides valuable prognostic information. Patients who achieve a complete pathological response after neoadjuvant chemotherapy are known to have significantly improved long-term outcomes. Conversely, poor responders face a higher risk of relapse and death. The identification of those subgroups that are more likely to benefit from breakthroughs in the personalised approach is a challenge of the current era where several targeted therapies are available. This review presents an overview of contemporary practice, and promising future trends in the management of early TNBC. Platinum chemotherapy, DNA damage response (DDR) inhibitors, immune checkpoint inhibitors, inhibitors of the PI3K-AKT-mTOR, and androgen receptor (AR) pathways are some of the increasingly studied therapies which will be reviewed. We will also discuss the growing evidence for less-developed agents and predictive biomarkers that are likely to contribute to the forthcoming advances in this field. Finally, we will propose a framework for the personalised management of TNBC based upon the integration of clinico-pathological and molecular features to ensure that long-term outcomes are optimised.

## 1. Introduction

Breast cancer (BC) is the most common cancer affecting women and is the leading cause of cancer-related death in women worldwide (1). Triple-negative breast cancer (TNBC), a highly heterogeneous subtype, represents approximately 15% of all breast cancers (2). TNBC behaves aggressively, has a poorer prognosis, and a higher risk of distant relapse and death relative to other BC subtypes (2). Genomic and transcriptomic data have enhanced our ability to understand the TNBC taxonomy and have enabled the identification of new therapeutic targets. The development of new therapeutic options and optimisation of personalised management strategies is critical in improving outcomes for affected patients.

This review provides an overview of contemporary practice in the treatment of early-stage TNBC and highlights promising future directions. We will discuss the growing evidence for newer therapies predicted to contribute to forthcoming advances in this field, and propose a framework for the personalisedmanagement of TNBC based upon the integration of clinical and molecular features.

## 2. Diagnosis and Clinical Presentation

TNBC is characterised by the absence of oestrogen (ER) and progesterone (PR) receptor expression, in addition to the absence of HER2 amplification as measured by immunohistochemistry or fluorescence *in situ* hybridisation. TNBC is disproportionately seen in younger women, as well as in Hispanic and African American populations (3). Disease-free intervals following primary treatment of early-stage (I–III) TNBCs are often short. The recurrence rate is 25%, with the highest risk of recurrence in the first three years after diagnosis and a median time to relapse after surgery of 18.8 months (4). Metastatic TNBC (mTNBC) exhibits a more aggressive phenotype than other BC subtypes, as demonstrated by a shorter chemotherapy response duration, and a shorter overall survival (OS) (median 13.3 months) (5).

## 3. TNBC Heterogeneity

TNBC is a heterogeneous disease with significant inter- and intra-tumour heterogeneity (6–8). Multiple efforts have focused on adequately addressing this biological complexity to enable the tailoring of therapeutic options to individual tumour characteristics.

### 3.1. Histological Subtypes

The current clinical definition of TNBC encompasses multiple histological subtypes. Approximately 85% of TNBCs are morphologically defined as invasive carcinoma of no special type (IC-NST). The remaining TNBCs are less common tumours of a special type, which are collectively associated with a poor prognosis (9). Individual special types display distinct pathological and molecular characteristics and prognoses. Tumours of indolent course include adenoid cystic, secretory and tubular carcinomas. Medullary histology is associated with a good prognosis and high response rates to chemotherapy, whereas metaplastic tumours show differentiation towards squamous epithelium with mesenchymal components and are frequently chemoresistant (10). An accurate histological examination marks the first step towards the identification of key mechanistic features that could be exploited for direct treatment (**Table 1**).

**Table 1:** Histological special subtypes of TNBC.

TNBC histopathological subtype	Key molecular features	Ref.
**Lobular**	Loss of E-cadherin expression and *CDH1* alterations.	(11)
	Enriched AR activity co-regulation and *FOXA1* network. Overexpression of genes under the control of *ESR1* and *PPARG*.	
	Frequent alterations in the PI3K network and *ERBB2*.	
	Recurrent *ESRRA* hotspot mutations.	
	Lower Ki67 index and lower expression of basal markers (CK5/6, *EGFR*, and *SOX10*) compared to IC- NST.	
**Metaplastic**	Increased frequency of mutations in the PIK3CA/AKT1/PTEN pathway compared to IC-NST. WTN pathway activation	(12)
**Medullary**	Predominant basal-like phenotype	(13)
	High frequency in *germline BRCA1* (gBRCA1) but rare in *germline BRCA2 (gBRCA2)* mutation carriers.	
	Prominent lymphoplasmacytic cell infiltrate in the tumour stroma and extensive intratumoural CD8+ TIL infiltration.	
**Apocrine**	Lower frequency of *TP53* mutations (25%) and *MYC* gains (0%) compared with IC-NST.	(14)
	High frequency of mutations in *PIK3CA* and other genes related to the PI3K signalling pathway (75%).	
**Adenoid cystic**	t (6,9)(q22–23;p23– 24). Fusion *MYB-NFIB*	(15)
	*MYBL1* rearrangements.	
	Low mutation rate.	
	Lack of high-level amplifications or homozygous deletions but recurrent 17q21-q25.1 gains and 12q12-q14.1 losses.	
	Lack of *TP53*, *PIK3CA* mutations. Recurrent mutations in *TLN2*, *MYB*, and *BRAF*	
**Secretory carcinoma**	t (12,15) (*ETV6; NTRK3)*	(16)
	Simple genomes with few CNA. Recurrent 8q, 1q, 16pq, and 12p gains, as well as 22q losses	

### 3.2. Molecular Subtypes

Numerous efforts to build upon the molecular classification of TNBCs have been proposed (**Table 2**). Here we review the most recognised classifiers that utilise genomic and transcriptomic data and summarise their predictive value when tested in early TNBC clinical cohorts. Many other classification approaches have been proposed (**Table S1**), with the absence of clinical evidence for treatment response limiting their use.

**Table 2:** Common TNBC Classification Methods.

Subtype	Main molecular characteristics	Biomarker value	Ref.
Classifier : Intrinsic subtypes*/ Hierarchical clustering , n = 825			
Basal-like	Expression of cytokeratin 5,6,17 typically expressed by the basal layer of the skin or airways. Very low level of expression of luminal-related genes. High frequency of TP53 and PIK3CA pathway activation (~9%) – via PTEN INPP4B. Cyclin E1 amplification and BRCA1 loss of function. Deregulation of the RB1 pathway, hyperactivation of FOXM1, MYC and HIF1-alpha/ARNT network hubs	Prognostic and predictive	(17)
Claudin Low	Low levels of cell adhesion proteins (claudin 3, 4, 7 and E-cadherin). Enrichment of mesenchymal traits and stem cell features Low to absent expression of luminal differentiation markers. High expression of stromal-specific and lymphocyte- or granulocyte-specific gene signatures		
**Lehmann et al. TNBCtype4/ k-means and consensus clustering , n = 2,347**			
Basal-like 1 (BL1)	Elevated cell cycle and DNA damage response (*CHEK1*, *FANCA*, *FANCG*, *RAD54BP*, *RAD51*, *NBN*, *EXO1*, *MSH2*, *MCM10*, and *RAD21)*	Prognostic and predictive (in vivo- clinical)	(18)
Basal-like 2 (BL2)	Enriched in growth factor signalling (*EGF, NGF, MET, Wnt/β-catenin*, and *IGF1R* pathways) and myoepithelial markers (*TP63* and *MME*)		
Immunomodulatory (IM)	Overexpression of genes encoding immune antigens and cytokine and core immune signal transduction pathways		
Mesenchymal (M)	Enriched in GE for EMT; cell motility (Rac1/Rho), ECM receptor interaction, and cell differentiation pathways (Wnt/β-catenin, ALK, and TGF-β).		
Luminal androgen receptor (LAR)	Activated androgen receptor (AR) signalling (DHCR24, ALCAM, FASN, FKBP5, APOD, PIP, SPDEF, and CLDN8)		
**Burstein et. al/Non-negative matrix factorization clustering , n = 198**			
Luminal androgen receptor (LAR)	Activation of *AR, ER*, prolactin, and ErbB4 signalling. RNA expression of *ESR1* and other oestrogen-regulated genes *(PGR*, *FOXA*, *XBP1*, and *GATA3*) in the absence of ER positivity by IHC. Focal gains on *CCND1*, FGF family and *MDGA2* and losses of 6q. *MUC1* overexpression	Prognostic	(19)
Mesenchymal (MES)	Dysregulated expression of genes involved in the cell cycle, mismatch repair, DDR networks, and hereditary BC signalling pathways. High expression of *OGN*, *ADIPOQ*, *PLIN1* and *IGF1.*		
Basal-like immunosuppressed (BLIS)	Low expression of molecules that control antigen presentation, immune cell differentiation, and innate and adaptive immune cell communication. High expression of SOX family transcription factors and *VTCN1*.		
Basal-like immune-activated (BLIA)	Upregulation of genes controlling B cell, T cell, and natural killer cell immune-regulating pathways, as well as activation of pathways mediated by *STAT* genes. *CDK1* amplification and overexpression of *CTL4*		
**FUSCC subtypes / k- means and consensus clustering , n =165**			
Immunomodulatory (IM)	High immune cell signalling and cytokine signalling gene expression. Activation of the adaptive immune system and INFg-related pathways. Overexpression of *ID01*	Prognostic and predictive (cell lines)	(20)
Luminal androgen receptor (LAR)	AR signalling. Low chromosomal instability. Increased frequency of *ERBB2* mutations. Enriched with Chr9p21 loss, decreased expression of *CDKN2A* and *E2F3*. Lower frequency of *RB1* losses/deletions and *CCND1* and *E2F3* gains/amplifications.		
Mesenchymal-like (MES)	Enriched in mammary stem cell pathways. Higher expression of JAK/STAT3 activation		
Basal-like and immune-suppressed (BLIS)	Upregulation of cell cycle, activation of DNA repair, and downregulation of immune response genes. High-HRD BLIS shows higher HRD scores irrespective of *gBRCA* status, higher proportion of Chr9p23 amp and Chr13q34 amp. Low-HRD BLIS is more likely to exhibit whole genome doubling.		
**Integrative Clusters/Integrative clustering framework (iCluster), n = 2,000**			
IntClust 4-	Flat copy number landscape and extensive lymphocytic infiltration. Strong immune and inflammation signature involving the antigen presentation pathway, *OX40* signalling, and cytotoxic T-lymphocyte-mediated apoptosis. Genomic copy number loss at *TCR* loci	Prognostic	(21, 22)
IntClust 10	Enriched within basal-like tumours. High-genomic instability, cis-acting alterations (5 loss/8q gain/10p gain/12p gain)		
**Prado-Vasquez Classification, n = 494**			
Cellular classification	Luminal (LAR): Lower activity in the nodes related to cell adhesion, G1/S transition of mitotic cell cycle and chemokine activity.	Pronostic (IM) and predictive (CLDN-high)	(23)
	Basal: Higher activity in cell adhesion and regulation of the actin cytoskeleton nodes.		
	Claudin-High (CLDN-high): Poor response to neoadjuvant chemotherapy. Higher activity in the chemokine activity functional node.		
	Claudin Low (CLDN-Low): Higher activity in the haptoglobin binding functional node and PPAR signaling pathway.		
Immune classification	Immune metanode (IM) positive		
	Immune metanode (IM) negative		

*Most prevalent intrinsic subtypes in TNBC listed.

#### 3.2.1. Intrinsic Subtypes

Breast cancers can be classified into six intrinsic molecular subtypes by gene expression (GE) profiling (17, 24) as follows: Luminal A, Luminal B, Her2 enriched, normal-like, basal-like, and Claudin low. Each subtype is identified within the TNBC group as defined by immunohistochemistry. Basal-like tumours are most frequent (50–75%). However, they are not exclusive to the TNBC phenotype (24). The claudin-low subtype represents 25–40% of TNBC and was more recently introduced (25).

Basal-like tumours are characterised by the presence of cytokeratins typically expressed by the basal layer of the skin, widespread genomic instability, high proliferation markers, loss of function of *BRCA1*, and dysregulation of *MYC* and *RB1* pathways (24). Claudin-low tumours have several features in common with basal-like tumours but are uniquely characterised by low levels of cell adhesion proteins, the enrichment of mesenchymal traits and stem cell features (26). Luminal tumours overexpress a ‘luminal signature’ containing *ESR1*, *GATA3*, *FOXA1*, *XBP1*, and *MYB*. Her2 amplification concomitantly with overexpression of *HER2*-amplicon-associated genes defines the Her2 enriched subtype (24).

Intrinsic subtypes provide independent predictive information regarding the response to neoadjuvant chemotherapy (NACT) when considering all subtypes of breast cancer, although not consistently for the TNBC cohort when viewed in isolation. Claudin-low tumours are associated with lower pathological complete response (pCR) rates compared to basal-like subtypes (27). In a subgroup analysis of the BrighTNess trial, pCR rates were higher for basal-like vs. non-basal tumours (52.3% vs 35.4%, p=0.003) (27). In contrast, no difference in pCR rate was observed with the addition of carboplatin for patients with basal-like TNBC vs non-basal TNBC in the CALGB40603 study (28). These results illustrate that the predictive value often linked to the basal-like subtypes has not always been reproduced in the early setting of TNBC, making intrinsic subtypes less reliable biomarkers of response within this group.

#### 3.2.2. Lehmann/Pietenpol Subtypes

Lehmann et al. selected clustering analyses to identify six TNBC subtypes displaying unique GE patterns and ontologies. Each subtype is characterised by the activation of specific signalling pathways that lead to a selective response to targeted therapies *in vivo* (18). Additional histopathological quantification and laser-capture microdissection prompted a refined classification with only four tumour-specific subtypes (TNBCtype-4). The original immunomodulatory and mesenchymal stem-like subtypes were deemed to originate from infiltrating lymphocytes and tumour-associated stromal cells, therefore excluding the impact of these elements from the classification. The new approach demonstrated differences in clinical baseline characteristics and both local and distant disease progression (29). Basal-like 1 (BL1) revealed increased markers of proliferation and elevated expression of the DNA damage response (DDR) genes. Basal-like 2 (BL2) is characterised by features of basal/myoepithelial origin and activation of growth factor pathways such as *EGF*, *NGF*, *MET*, *Wnt/β-*catenin, and *IGF1R*. The mesenchymal (M) subtype displays activation of pathways involved in epithelial–mesenchymal transition (EMT), cellular differentiation, and growth pathways. Luminal androgen receptor (LAR) tumours are characterised by high expression of androgen receptor (*AR*) and downstream AR targets, and enrichment of pathways involved in steroid synthesis, porphyrin metabolism, and androgen/oestrogen metabolism (18).

A retrospective analysis from the validation cohort of the TNBC subtype classification presented by Masuda et al. showed that the likelihood of pCR with NACT was subtype dependent. BL1 had the highest pCR rate; BL2 and LAR had the lowest. TNBC subtypes demonstrated improved pCR predictions compared to intrinsic subtypes (basal-like vs. non-basal) (30). In a retrospective analysis of clinically annotated microarray datasets of BC patients, TNBC type-4 subtyping was not associated with significant differences in pCR in the TNBC subgroup. However, the overall incidence of pCR for the subtypes demonstrates trends similar to those observed in previous studies. BL1 displayed the greatest pCR rate (41%), and LAR and BL2 displayed the lowest (29 and 18%, respectively). BL1 patients had significantly higher pCR rates compared with other subtypes (49% vs. 31%, p = 0.04) (29). Santonja et al. explored the performance of Lehmann subtypes and their association with pCR in 125 TNBC patients treated with neoadjuvant anthracyclines and/or taxanes with and without carboplatin, and their results agreed with previous reports (31). The pCR rate for carboplatin containing regimens was highest for BL1 tumours (80% vs 23%, p = 0.027). LAR tumours had the lowest pCR rate for all treatments (14.3% vs 42.7%, p = 0.045).

#### 3.2.3. Burstein Subtypes

Burstein and colleagues applied non-negative matrix factorisation clustering to identify four distinct TNBC subtypes characterised by key molecular features and prognosis: LAR, mesenchymal, basal-like immunosuppressed (BLIS), and basal-like immune-activated (BLIA). BLIS and BLIA had the best and worst clinical outcomes, respectively. LAR and mesenchymal subtypes revealed significant overlap with Lehmman’s classification. Burstein’s subtypes based on immune signalling (BLIA, BLIS) revealed a combination of BL1 and BL2 subtypes (19).

#### 3.2.4. FUSCC Classification

Liu et al. developed a classification system based on the transcriptome profiles of both messenger RNAs and long non-coding RNAs to divide TNBC into four distinct clusters. Cluster A: immunomodulatory subtype, Cluster B: luminal androgen receptor subtype (LAR), Cluster C: mesenchymal-like subtype, and Cluster D: basal-like and immune-suppressed (BLIS) subtype. No significant difference in prognosis was found between the four subtypes. Tumours classified as the BLIS subtype experienced poorer relapse-free survival (RFS) compared with all other subtypes (20, 32). Further classification of BLIS tumours based on their homologous recombination deficiency (HRD) status (33) showed that high-HRD BLIS TNBCs and low-HRD BLIS TNBCs exhibited distinctive genomic characteristics and prognoses. Patients with tumours defined as low-HRD had a worse prognosis than those in the high-HRD subgroup (5-year RFS of 73 and 95%, respectively, p = 0.002) (32).

#### 3.2.5. Integrative Clusters

Combining GE and DNA copy number analysis within the METABRIC dataset further expanded the taxonomy of breast cancer (22). Eleven Integrative Clusters (IntClust) with distinctive copy number profiles and clinical outcomes were identified. TNBCs are most frequently classified as IntClust 4ER- or IntClust 10. Rueda et al. showed that patients with tumours classified as IntClust 10 (n = 222) have a low probability of late relapse (five years after diagnosis), while those classified as IntClust4ER- (n = 73) show a persistent and increasing risk of relapse or cancer-related death after 5 years. Classification by immunohistochemistry or intrinsic subtypes did not show this risk (21). The predictive value of IntClust to define response to NACT is yet to be fully established.

#### 3.2.6. Prado-Vasquez Classification

Prado-Vasquez et al. developed a probabilistic graphical model to classify the cellular components of tumours into four groups based on the ‘stem cell hypothesis,’ defined based on the grade of development of the cells from which they derived luminal (LAR), basal, claudin-high (CLDN-high), and claudin-low (CLDN-low). The sparse k-means method was used to define high or low immune activity and to classify the tumour as immune metanode positive or negative. Immune metanode activity was prognostic overall, and particularly in the luminal group defined by the cellular classification and TNBC type4-LAR (23).

Combining molecular knowledge with patient management is an increasingly accepted technique across tumour types. In early TNBC, lack of reproducibility and the absence of a unified approach have led to the continuous use of unselected clinical strategies that remain insufficient. Stable commonalities among the classification methods of molecular subtyping in TNBC suggest the presence of clear biological groups suitable for personalised therapeutic interventions. For instance, luminal-like and mesenchymal tumours are consistently identified across the methods with decent overlap and reproducible outcome data. Moreover, most methods include a measurement of the interaction between tumours and the immune response, highlighting the importance of considering this element as a key component of the TNBC taxonomy. Overall, these efforts provide the basis for understand how the molecular complexity of TNBC influences outcomes. Considering the treatment response due to dynamic network interaction, rather than focusing on individual static components, is likely to have more predictive power. But even with reproducible and reliable classification, delivering this in a clinical timeframe suitable for neoadjuvant therapy decision-making remains a challenge.

**Summary Box 1—Biological and clinical features of TNBC**

–TNBC is characterised by the absence of ER, PR and HER2 expression and is associated with high early response rates to treatment and poor prognosis.
–TNBC is a heterogeneous disease with a high level of inter and intra tumour heterogeneity.
–Multiple TNBC classifications that split TNBC tumours based on unique molecular features have been described but have yet to be incorporated into routine clinical practice.

## 4. Overall Approach to the Treatment of Early Stage TNBC

Therapeutic options for early TNBC have traditionally been limited to cytotoxic chemotherapy, surgery, and radiotherapy. Significant advances in basic and clinical research have led to tangible improvements in the current therapeutic arsenal. The FDA has now approved pembrolizumab immunotherapy for use along with chemotherapy for high-risk early-stage TNBC following survival data from the KEYNOTE-522 trial (34). This has established immunotherapy as a new standard of care in the United States, and it is anticipated to reach clinical practise in other countries in the near future. Similarly, the recent FDA approval of Olaparib for the adjuvant treatment of high-risk germline *BRCA* (*gBRCA*) carriers following results of the OlympiA trial is expected to reshape clinical practice (35). These encouraging developments highlight the importance of a personalised treatment approach and focus attention on the unresolved challenges of appropriate patient selection and derived toxicity.

Closing the gap between preclinical advances and the clinical setting remains a prolonged and challenging process.

### 4.1. (Neo)adjuvant Chemotherapy

The effect of polychemotherapy compared with no chemotherapy across all BC subtypes was assessed as part of the 2012 Early Breast Cancer Trialists’ Collaborative Group (EBCTCG) meta-analysis of 32,000 patients. This resulted in a ~50% reduction in 2-year recurrence and a 20–25% decrease in BC (36). Chemotherapy is particularly important in managing TNBC as these tumours demonstrate a better response compared to other subtypes of BC and the importance of achieving and optimising the early treatment response in these tumours is well recognised.

#### 4.1.1. Anthracyclines

Anthracyclines target cell proliferation pathways by interacting with DNA gyrase and leading to DNA double-strand breaks (DSBs). The ABC trials proved that the addition of an anthracycline to taxane and cyclophosphamide improved patient outcomes, with the greatest benefit in high-risk patients, those with lymph node involvement or hormone-negative disease (37). More recently, a large meta-analysis by Braybrooke et al. found an 18% reduction in the 10-year recurrence risk with the addition of anthracycline to taxane chemotherapy, as compared to taxane alone (38). There are multiple anthracycline-taxane-based regimens now in use, with evidence to support one “optimal” standard of care regimen for TNBC lacking (39).

Anthracycline-free chemotherapy regimens are considered when cardiotoxicity is a concern, and routine use of such regimens for treatment de-escalation is an area of increasing interest (40) (see **Table 3**). Evidence regarding efficacy as a standard treatment for TNBC is conflicting, although a recent meta-analysis has established anthracycline-free chemotherapy to be acceptable for lower risk, early-stage HER2-negative BC (39).

**Table 3:** Major clinical trials evaluating adjuvant anthracycline-free chemotherapy regimens for patients. with stage I-III TNBC.

Trial	Phase	Disease Setting	TNBC sample size	Treatment	Primary endpoint	Results (ITT population)	Ref.
ABC – joint analysis:(USOR 06-090, NSABP B-46-I/USOR 07132, and) NSABP B-4)	3	Adjuvant treatment of HER2–negative breast cancer	31% of patients (n = 4,156)	Docetaxel/ cyclophosphamide (TC) vs doxorubicin/cyclophosphamide/taxane (TaxAC)	iDFS	4y IDFS ITT Population 88.2% vs 90.7% (P = .04) TNBC Node negative 87% vs. 89.5% (HR 1.31 95% CI 0.86–1.99) 1-3 positive nodes. 74.6% vs 85.5% (HR 1.58 95% CI 0.90–2.79) >=4 positive nodes 60.8% vs. 71.8% (HR 1.34 0.62–2.91)	(37)
MASTER	3	Adjuvant Treatment of high-risk HER2-negative operable breast cancer	120	Docetaxel/ cyclophosphamide (TC) vs cyclophosphamide/epirubicin/fluorouracil followed by docetaxel (CEF-T) vs epirubicin and cyclophosphamide followed by paclitaxel (EC-P)	DFS	5-year DFS CEF-T versus EC-P, 85.1% vs. 85.9% (HR = 0.99 90% CI: 0.75–1.30, non-inferior P = 0.045). TC versus EC-P 85% vs 85.9% (HR 1.05 90% CI 0.79–1.39, non-inferior P = 0.048	(41)
DBCG 07-READ	3	Adjuvant treatment in high risk TOP2A-normal breast cancer	459	Epirubicin/cyclophosphamide followed by docetaxel (EC-T) Vs docetaxel/ cyclophosphamide (TC)	DFS	5y DFS 87.9% vs. 88.3% (HR, 1.00 95% CI, 0.78–1.28)	(42)
HORG	3	Adjuvant treatment of HER2-negative invasive BC and at least one positive axillary lymph node	74	Dose-dense epirubicin/5-fluorouracil/cyclophosphamide followed by docetaxel (FEC-D) Vs docetaxel/ cyclophosphamide (TC)	DFS	3y DFS: 89.5% vs 91.1% (HR 1.147, p = 0.568)	(43)
WGS Plan B + Success C Pooled Analysis	3	Adjuvant	Not available	Docetaxel/ cyclophosphamide (TC) vs epirubicin/cyclophosphamide followed by docetaxel (EC-T) or epirubicin/5-fluorouracil/cyclophosphamide followed by docetaxel (FEC-D)	DFS	ITT DFS HR = 1.04 (95% CI: 0.85 – 1.19, p = 0.96) pN2-3 patients HR 0.69 (95%-CI 0.48– 0.98, p = 0.04) TNBC DFS HR = 0.99( 95% CI 0.76 – 1.30, p = 0.95)	(44)

#### 4.1.2. Microtubule Targeting Agents

Taxanes inhibit cell division by stabilising microtubules, preventing depolymerisation, spindle formation, and progression through the cell cycle. Paclitaxel and docetaxel are regularly used to treat early-stage TNBC. An EBCTCG meta-analysis showed that the addition of taxane to anthracycline resulted in a proportional reduction in mortality rates of 15–20% (36). The European Cooperative Trial in Operable Breast Cancer (ECTO) also demonstrated significant improvements in RFS and distant RFS (45). Although this evidence is not unique to TNBC, these studies provide the strongest evidence to support taxane use in this cohort. BL1 and BL2 tumours appear to derive an increased benefit from this drug class (46).

There are several novel alternatives to traditional taxanes under investigation. Nab-paclitaxel is a solvent-free albumin-bound nanoparticle formulation of paclitaxel. It potentially enables higher intra-tumoural taxane concentrations, better efficacy, and improved tolerability. The GeparSepto (47) and ETNA trials (48) showed conflicting results with a significant difference in pCR rates seen only in GeparSepto (**Table S2**), which may reflect the relative dose intensities used.

Epothilones are promising alternatives to taxanes in development. These novel potent microtubule stabilisers can bypass common resistance mechanisms seen with taxanes, such as drug efflux pumps and β-tubulin. In the early setting, the phase 3 TITAN trial has shown similar efficacy and reduced rates of peripheral neuropathy, dose modifications, and discontinuation with Ixabepilone comparised with paclitaxel (49).

#### 4.1.3. Platinum Salts

The clinical activity of platinum agents has been significantly associated with a DDR vulnerability in both sporadic and *gBRCA*-associated TNBC. Carboplatin is increasingly used in neoadjuvant regimens, improving both pCR and long-term outcomes (50). Please see section *DNA Damage Response (DDR)*.

#### 4.1.4. Capecitabine

Capecitabine is an oral prodrug of the antimetabolite 5-fluorouracil. Capecitabine is not currently recommended in clinical guidelines for the neoadjuvant or adjuvant treatment of TNBC, though it is selectively used as a post-neoadjuvant treatment for residual TNBC. Insights for use in the adjuvant setting are accumulating (**Table 4**), but in most cases, studies have not incorporated the molecular features of the TNBC cohort into the planned analysis for response assessment. The recent phase 3 CBCSG-010 trial for unselected patients with TNBC with concomitant use of capecitabine 1,000 mg/m^2^ and standard anthracycline-taxane adjuvant chemotherapy (ACT) established a significant 5-year disease free survival (DFS) benefit (53). This is supported by the FINXX trial (54) and the Ye et al. meta-analysis, which demonstrated improved DFS and OS with a tolerable increase in toxicity (56).

**Table 4:** Major clinical trials evaluating capecitabine in patients with stage I–III TNBC.

Trial	Phase	Disease Setting	TNBC sample size	Treatment	Primary endpoint	Results	Ref.
						(TNBC cohort)	
CREATE-X	3	Adjuvant treatment of residual HE2 negative early-stage BC following Taxane &/or anthracycline based NACT	286	6–8 cycles of capecitabine vs control	DFS	5y DFS: 70% vs 56% (0.58; 95% CI, 0.39 to 0.87) 5y OS: 79% vs. 70% (0.52; 95% CI, 0.30 to 0.90)	(51)
GEICAM/2003- 11_CIBOMA/2004-01	3	Neoadjuvant or adjuvant treatment of early-stage TNBC following Taxane &/or anthracycline based NACT/ACT	876	8 cycles of extended capecitabine after standard chemotherapy vs. observation	DFS	5y DFS: 80% vs 77% (HR, 0.82; 95% CI, 0.63 to 1.06; P = .136) 5y OS: 86.2% vs 85.9 (HR, 0.92; 95% CI, 0.66 to 1.28; P = .623)	(52)
CBCSG-010	3	Adjuvant treatment of early stage TNBC	585	standard anthracycline-taxane chemotherapy with or without 3 cycles of capecitabine	5y DFS	86.3% v 80.4%; HR 0.66; 95% CI, 0.44 to 0.99; P = .044	(53)
FinXX Trial	3	Adjuvant treatment of early stage breast cancer	202	Docetaxel, Epirubicin, and Cyclophosphamide chemotherapy with or without 3 cycles of capecitabine	RFS	HR, 0.53; 95% CI, 0.31–0.92; P = .02	(54)
SYSUCC-001	3	Adjuvant treatment of early-stage TNBC following standard adjuvant therapy	434	1 year of capecitabine vs observation	DFS	5y DFS: 83% vs 73% (HR 0.64 95% CI, 0.42–0.95 P = .03)	(55)

### 4.2. Bone Modifying Agents

Adjuvant bisphosphonates are recommended for breast cancer patients with low-oestrogen status at high risk of relapse to decrease skeletal metastases and improve OS and DFS, as evidenced by the AZURE trial and the EBCTCG meta-analysis, both of which included patients of all BC subtypes (57, 58). While most of the evidence for bone modifying agents in TNBC comes from studies of patients receiving ACT, benefit is also probably derived in the neoadjuvant setting (59). A subgroup analysis of patients receiving neoadjuvant ZA alongside NACT in the AZURE trial led to improved pCR rates (60). The role of RANK-L remains under investigation. The D-CARE trial of adjuvant denosumab showed no improvement in bone metastasis free survival, invasive disease free survival (iDFS), or OS in high-risk early breast cancer. This suggests that the mechanisms by which bisphosphonates act against the metastatic potential of BC cells are broader and more sustained than the known effects on bone cell function (61).

**Summary Box 2—Standard of care treatments in TNBC**

−Sequential anthracycline-taxane based regimens are considered standard of care.
−Anthracycline-free chemotherapies are considered for lower risk tumours or in patients where cardiotoxicity is a concern.
−Taxane-free chemotherapy or use of an alternative microtubule stabiliser is considered in patients with peripheral neuropathy or taxane hypersensitivity reactions.
−Bisphosphonates are recommended for the treatment of operable breast cancer of all subtypes in patients with low oestrogen states, whether natural or induced. They should particularly be considered in patients at high risk of relapse or treatment-related bone loss.

### 4.3. Treatment Schedule

#### 4.3.1. Neoadjuvant vs. Adjuvant Chemotherapy

Chemotherapy can be delivered in an adjuvant or neoadjuvant setting with no significant difference in long-term outcomes, as illustrated by the NSABP B-18, EORTC 10902, and IBBGS trials (62–64). More recently, an EBCTCG meta-analysis demonstrated no significant difference in distant recurrence or death between NACT and ACT but a more frequent local recurrence rate (65). A TNBC-specific meta-analysis suggested that NACT is associated with a comparable DFS but worse OS than ACT (66), perhaps explained by patients with higher disease burden being more likely to receive NACT. In this meta-analysis, patients who achieved pCR had superior OS and DFS compared to those treated with ACT. This evidence does not support the suggestion that NACT promotes cancer cell dissemination (67).

Advantages of NACT include downstaging tumours, resulting in increased rates of breast-conserving surgery and associated improved cosmesis and reductions in postoperative lymphoedema. Additionally, it allows the assessment of treatment response, provides valuable prognostic information (68), guide choice of post-surgical treatment and allows for ineffective treatment to be ceased to avoid unnecessary toxicity. NACT also provides an ideal platform for translational research, assessment of biomarkers, and genetic testing (69).

The same anthracycline/taxane-based regimens are typically used in NACT and ACT. Whether the scheduling of these combinations has any effect on efficacy has been the subject of extensive research. It is been shown that using taxanes and anthracyclines sequentially increases efficacy and decreases toxicity (70). Some evidence suggest that administration of taxane chemotherapy before anthracyclines is associated with improved pCR rates (71).

#### 4.3.2. Dose-Dense and Metronomic Chemotherapy

There has been an increasing interest in personalising the treatment timetables to take patient and tumour characteristics into account. Dose-dense NACT is now a widely accepted treatment strategy for high-risk TNBC in order to prevent cancer cell repopulation (72). It has been consistently shown to improve the rates of pCR, breast-conserving surgery, and recurrence in hormone-low BC (73, 74). Although this regimen has not translated into a significant survival benefit (74), this approach should be considered in selected patients with a high disease burden. Dose-dense ACT improves DFS and OS rates in patients with low hormone receptor levels, although this is accompanied by increased toxicity and patients need to be selected carefully (75).

At the other end of the spectrum, metronomic chemotherapy is given at a minimum biologically effective dose either continuously or with minimal extended breaks from treatment to reduce severe toxicity. It is thought to have angiogenic, stroma-targeting, and immunostimulatory effects (76). It has been investigated as a single approach and is being used in combination to intensify standard chemotherapy. It may have a role as a maintenance therapy for high-risk patients or for use by patients who would not otherwise be able to tolerate the adverse effects of standard treatments. The SYSUCC-001 study showed significant improvement in 5-year DFS with 1 year of maintenance capecitabine (55). The IBCSG 22-00 trial confirmed a 7.9% reduction in the absolute risk of relapse in patients with node-positive TNBC (77) after 1 year of low-dose capecitabine and methotrexate maintenance treatment, although no improvement in DFS was observed.

### 4.4. Assessing Response to NACT

Residual Cancer Burden (RCB) classifies tumour response to chemotherapy using a numeric score based on four characteristics of surgical outcome: primary tumour bed dimensions; cellularity fraction of invasive cancer; size of largest metastasis; and number of positive lymph nodes (68). Four prognostic categories were established (**Table 5**). It has been shown that NACT achieves a pCR in slightly over a third of patients with TNBC, and these patients enjoy excellent long-term survival outcomes (78, 79). Higher rates of pCR following NACT are seen in TNBC as compared to other subtypes, despite the high rate of disease relapse in this cohort. This is believed to derive from poor outcomes in patients with residual chemotherapy-resistant disease (80). RCB after NACT can accurately predict both event-free survival (EFS) and DFS and is commonly used as a surrogate outcome in clinical trials (79).

**Table 5:** Residual cancer burden categories.

RCB-0	‘Pathological complete response (pCR)’ defined by the absence of tumour cells in breast and axilla
RCB-I	‘Minimal residual disease’
RCB-II	‘Moderate residual disease’
RCB-III	‘Extensive residual disease

Liquid biopsies for circulating tumour DNA (ctDNA) measurement are a promising dynamic approach to assess residual disease and predict treatment response in real-time (81). Fragments of DNA released by apoptosed or necrosed tumour cells can be longitudinally measured in the blood samples of patients. Detection of high ctDNA levels at the time of surgery has been associated with reduced DFS and OS rates, and clearance of ctDNA during NACT has been associated with improved outcomes across all BC subtypes (82). Clinical trials that incorporate this approach for patient selection are imminent.

### 4.5. Post Neoadjuvant Treatments

Patients with residual disease after surgery are often considered for further systemic therapy. Current treatment options in this setting following NACT include capecitabine and poly ADP-ribose polymerase inhibitors (PARPi) for g*BRCA* carriers.

The Create-X trial demonstrated that six to eight cycles of capecitabine improved 5-year DFS and OS compared to no further therapy, especially in the TNBC cohort (51). In contrast, the GEICAM/2003-11_CIBOMA/2004-01 trial failed to show a statistically significant increase in DFS with the use of eight cycles of adjuvant capecitabine. Of note, a pre-planned analysis of this study showed that the non-basal TNBC cohort derived the most benefit from receiving capecitabine (52). Significant differences in study populations limit direct comparisons between these two studies. Create-X enrolled an Asian population who are highly efficient metabolizers of fluoropyrimidines, all of whom had high-risk pathologically-assessed residual disease. In contrast, GEICAM/CIBOMA accrued patients from Europe and South America, only 80% of whom had residual disease. Meta-analyses on the topic have concluded upon an overall improvement in DFS and OS with capecitabine (83) and opinions from the St. Gallen International Conference found 87% of experts would offer capecitabine to patients with residual TNBC in the post-neoadjuvant setting (84). Differences in outcomes at a population level and issues with toxicity have led to capecitabine being offered on a case-specific basis rather than as a standard of care (85). The GEICAM/CIBOMA data indicate that more detailed investigation is needed into exactly which TNBC sub-types would benefit from capecitabine.

The OlympiA trial recruited 1,836 patients with HER2-negative cancers, 82% classified as TNBC, and showed that 52 weeks of adjuvant olaparib was associated with a significant DFS improvement in patients with g*BRCA1/2* mutations (3-year iDFS of 85.9% for olaparib vs 77.1% for placebo) (35). A 32% reduction in the risk of death versus placebo (HR = 0.68; 95% CI 0.50–0.91; p = 0.0091) led to the recent FDA approval for olaparib in this setting.

The optimal treatment for residual disease after NACT remains a matter of debate, particularly for g*BRCA* carriers with high-risk TNBC. A direct comparison between adjuvant olaparib and capecitabine is unavailable. The theoretical advantage for olaparib use includes targeting a known tumour susceptibility in a selected population, leading to an improved response and improved tolerability compared to standard cytotoxics. Interestingly, a phase 2 trial that assessed the value of molecularly targeted postneoadjuvant treatment vs choice of clinician in TNBC patients with residual disease did not demonstrate the superiority of this approach (86). Despite the limitations with regard to the primary outcome, an example was set for biomarker-driven clinical trials and the use of ctDNA in optimising the selection of biomarker-treatment partners. Patient preference and financial issues clearly need to be considered in this setting.

**Summary Box 3—Key concepts in the current treatment of TNBC**

−Chemotherapy can be given in the adjuvant or neoadjuvant setting and the same regimens are typically used. Long-term survival outcomes are similar.
−Advantages of NACT include a rapid evaluation of tumour response, prognostication using RCB scoring, and improved surgical outcomes.
−RCB is strongly associated with long-term outcomes in TNBC.
−Patients with TNBC who are at increased risk of relapse after chemotherapy in the neoadjuvant setting benefit from adjuvant capecitabine. Patients in the g*BRCA* subgroup benefit from PARP inhibitors.
−Sequential liquid biopsies to assess ctDNA levels represent a possibility for monitoring treatment response in real-time.

## 5. Targetable Molecular Pathways

Much progress has been made in defining and treating TNBC according to aberrations on the molecular level, although the derivation and use of biomarkers to select patients for specific treatments has been somewhat lacking. To make further progress, the identification of predictive biomarkers must be a central focus of our research and, once secured, used to guide and to select patients most likely to derive benefit from targeted treatments.

**Tables S3–S5** summarise ongoing trials contributing to the use of molecularly targeted treatments for early TNBC.

### 5.1. DNA Damage Response (DDR)

TNBCs are frequently deficient in DDR pathways and exhibit high chromosomal instability (7, 87). The repair of DNA double-strand breaks (DSBs) relies on the homologous recombination (HR) pathway. Dysfunctional activity of genes involved in this process compromises the ability of cells to mend DSBs, thereby inducing Homologous Recombination Deficiency (HRD) (88).

HRD can occur *via* numerous mechanisms, all resulting in similar phenotypic and genotypic features to those of *BRCA* mutant tumours, an observation termed ‘*BRCA*-ness’. Phenotypic and molecular similarities between *BRCA-*associated BC and sporadic TNBC have led to the application of similar therapeutic interventions in both groups. In patients with *BRCA* mutations and *BRCA*ness features, a compromised DDR pathway facilitates increased sensitivity to drugs such as platinum and PARPi, based on the concept of synthetic lethality (89).

Approximately 10–20% of TNBC harbour g*BRCA* mutations, and 70% of *gBRCA1* and 16% of *gBRCA2*-associated tumours are classified as TNBC (90). Somatic *BRCA* mutations are uncommon in sporadic TNBCs (6, 24, 32). *BRCA*1 and 2 mutations, and hypermethylation of the *BRCA*1 promoter, only account for some TNBCs that exhibit functional evidence of HRD. In total, around 40% of BCs are identifiable as HRD in the absence of these changes (91). Dysfunctional *BRCA* pathways are frequently enabled by other mechanisms; for example, *RAD51* and *PALB2* mutations can confer a *BRCA*-ness phenotype (91).

#### 5.1.1. Therapeutic Approaches

##### 5.1.1.1. Platinum Agents

The cytotoxic activity of platinum is mediated by the formation of platinum–DNA adducts that interfere with DNA replication and transcription, activating DNA-damage recognition and repair, cell-cycle arrest, and apoptosis.

Platinum-containing regimens have not been regarded as the standard of care for treating TNBC in most guidelines to date. Several trials have investigated the addition of platinum agents to standard chemotherapy for this subgroup based on the potential-increased susceptibility of TNBC to DNA-damaging compounds (30) (**Table 6**). Improved pCR rates with the addition of carboplatin have been a consistent finding, with confirmed EFS benefit in two large randomised studies, GeparSixto and BrighTNess (92–95, 101–103). These results have led to the inclusion of carboplatin within neoadjuvant regimens for high-risk TNBC in the American Society of Clinical Oncology guidelines.

**Table 6:** Major clinical trials involving platinum agents in patients with stage I–III TNBC.

Trial	Phase	Disease Setting	TNBC sample size	Treatment	Primary endpoint	Results	Ref.
CALGB 40603	2	Neoadjuvant - Stage II to III TNBC	443	Addition of carboplatin and/or bevacizumab to neoadjuvant paclitaxel followed by dose dense doxorubicin/cyclophosphamide (ddAC)	pCR	pCR 54% vs 41%. (p = 0.0029) No EFS or OS benefit	(92)
BrighTNess	3	Neoadjuvant -Stage II o III TNBC	634	Addition of carboplatin and/or veliparib to neoadjuvant paclitaxel followed by doxorubicin/cyclophosphamide (AC)	pCR	Carboplatin Arm - pCR 58% vs 31%. (p = 0.0001).EFS (HR: 0.57 CI 0.36−0.91, p = 0.018) Veliparib+carboplatin Arm pCR 53% vs. 31%: EFS (HR 0.63 CI 0.43−0.92)	(93)
GeparSixto	2	Neoadjuvant- Stage II to III HER+ and TNBC	315	Paclitaxel, doxorubicin, and bevacizumab with or without carboplatin	pCR	pCR 53.2% vs 36.9%( p 0.005) DFS (HR 0.56 CI 0.34–0.96; p = 0.022) No OS benefit	(94)
Byrski et al.	2	Neoadjuvant - *BRCA1* associated BC	107	Single-agent cisplatin	pCR	pCR 61%	(95)
INFORM	2	Neoadjuvant - Stage I – III BRCA carriers/ HER2 negative BC	118	Single-agent cisplatin vs doxorubicin-cyclophosphamide (AC)	pCR	pCR 23% vs 29% (RR of 0.70, 90% CI, 0.39 to 1.2) RCB0/1 36% vs 47% (RR, 0.73; 90% CI, 0.50 to 1.1)	(96)
ECOG-ACRIN EA1131	3	Adjuvant/Post NACT - Residual disease, stage II-III basal-like TNBC	562	Platinum vs capecitabine vs observation	iDFS	3y iDFS – 42% vs 49% (1.06 (95% RCI, 0.62 to 1.81).	(97)
PATTERN	3	Adjuvant - TNBC	647	Carboplatin and paclitaxel vs cyclophosphamide, epirubicin, and fluorouracil followed by docetaxel	DFS	DFS 86.5% vs 80.3% (HR 0.39 (95% CI, 0.15-0.99; P = .04)	(98)
NCT03301350	2	Neoadjuvant - TNBC	29	Carboplatin/paclitaxel followed by dose-dense doxorubicin/cyclophosphamide	pCR	pCR = 33%	(99)
PARTNER	2/3	Neoadjuvant - TNBC and/or gBRCA associated Her2 neg BC.	527	Paclitaxel/carboplatin with or without olaparib followed by anthracycline-based chemotherapy	Safety pCR	Pending	(100)
NCT03876886	3	Adjuvant- High-risk node-negative or node-positive TNBC with HRD	200	Dose-dense AC-Tvs TP	3y DFS	Pending	
NCT04664972	2	Neoadjuvant -Operable TNBC	166	Docetaxel/cisplatin (TP) vs docetaxel/doxorubicin/cyclophosphamide (TAC)	pCR	Pending	
NCT03168880	3	Neoadjuvant - Large Operable or Locally Advanced TNBC	720	Paclitaxel with or without carboplatin followed by anthracycline-based chemotherapy	DFS OS	Pending	

Combining carboplatin with anthracycline/taxane NACT increases haematological and gastrointestinal toxicity, which in turn has implications for patient selection. Predictive biomarkers to identify those patients deriving the most benefit from the addition of platinum, for example, *gBRCA* mutations, have been investigated. Single-agent cisplatin has shown conflicting results for *BRCA* carriers (96). The PARTNER (NCT03150576) trial includes a cohort of *gBRCA*-mutated patients (100) and will help elucidate the effect of platinum and PARPi in this subgroup.

There is currently no routine indication for platinum agents in the post-neoadjuvant setting. The EA1131 study (NCT02445391) was closed early as neither cisplatin nor carboplatin was able to demonstrate non-inferiority or superiority over capecitabine, and toxicity rates were higher (97).

##### 5.1.1.2. PARP Inhibition

Poly ADP-ribose polymerase (PARP) activity is crucial for maintaining the correct fork speed and fidelity of DNA synthesis. PARP1 is involved in the response to single-strand DNA (ssDNA) damage and maintains genome integrity *via* base excision repair. PARP1 is also a critical early event for DNA DSB repair activation and regulation of resection (104). PARP inhibition causes replication stress, induces ssDNA breaks and affects the normal regulation of p53 and its downstream effectors (105). In tumours that have deficiencies in the HR pathway, the accumulation of DSBs originating from primary ssDNA breaks leads to cell cycle arrest and death (106).

Robust evidence now supports the efficacy of single-agent PARPi in BC patients with g*BRCA* mutations who have received prior chemotherapy (107, 108). A variety of PARPi and combinations have now been explored in both patients with g*BRCA* mutations and sporadic (non-*BRCA*) TNBC in the early setting.

Evidence to date for the use of olaparib is promising, both as monotherapy and in combination with chemotherapy, immunotherapy, or radiotherapy. In the neoadjuvant setting, olaparib was given as monotherapy in 32 patients with unselected TNBC for up to 10 weeks before chemotherapy (109) with an overall objective response rate of 56.3% vs 51.9% among patients not harbouring *gBRCA1*/2 or germline *PALB2* mutations. A numerical enrichment of somatic HR mutations and *BRCA*1 methylation in the responding group suggests favourable activity of olaparib here. Other trials in the neoadjuvant setting combine olaparib with chemotherapy. GeparOLA included patients with HER2-negative BC and HRD, received paclitaxel with olaparib or carboplatin followed by epirubicin and cyclophosphamide (110). No formal testing between the arms was planned but increased benefit from olaparib was observed in young (<40 years) and HR-positive patients. In the TNBC subgroup, the pCR rate was 56.0% with olaparib and 59.3% with carboplatin. PARTNER is a phase 3 trial that assesses the addition of olaparib to neoadjuvant platinum-based chemotherapy in the treatment of TNBC and g*BRCA*-derived tumours. Preliminary safety results show that the combination of olaparib and platinum has an acceptable and manageable toxicity profile (111). In the I-SPY2 trial, research arm patients received olaparib and Durvalumab with paclitaxel, then doxorubicin and cyclophosphamide (112), which increased pCR in the TNBC group (27–47%). Immune-rich tumours had greater sensitivity to this treatment. The adjuvant phase 1 RadioPARP trial for patients with inflammatory, locoregionally advanced or mTNBC, or patients with residual disease after surgery for TNBC, sought to evaluate safety and dosing for olaparib in combination with radiotherapy (113). Olaparib was escalated to the maximum target dose of 200 mg twice daily with no dose-limiting toxicity.

Talazoparib has been reviewed in the neoadjuvant setting as monotherapy and along with chemotherapy. TALA was a pilot study that recruited 20 patients with operable BC and a *BRCA* mutation to receive talazoparib monotherapy for 6 months (114). Despite the small sample size, this trial showed an encouraging pCR rate of 53% and RCB-0/I of 63%, with a manageable safety profile. In the I-SPY2 trial, talazoparib combined with irinotecan for HER2-negative patients had limited activity beyond that seen with standard treatment (115).

Veliparib has also been evaluated in the neoadjuvant setting in the I-SPY2 trial (116). The addition of veliparib to carboplatin-containing chemotherapy increased the pCR rate in the TNBC group from 26 to 51%. This combination was further assessed in the phase 3 BrighTNess trial in 634 patients with TNBC (94), where no additional benefit for veliparib above that achieved by adding carboplatin, regardless of *BRCA* mutation status, was found. A key limitation of this study is the low dose of veliparib, less than half of that used in the BROCADE-3 study in the advanced disease setting (117). Veliparib has been combined with radiotherapy for inflammatory or locoregionally recurrent TNBC, which results in significant local toxicity (118).

Both talazoparib and olaparib are effective as monotherapies in patients carrying g*BRCA* mutations. Given the low dose of velaparib used in the BrighTNess trial, and considering individual PARPi differences in PARP trapping capacity, the potential summative benefit from the addition of platinum to PARPi cannot be excluded. This encourages further investigation into the role of other PARPi such as olaparib and talazoparib and the great potential for combination therapy, as demonstrated by the ongoing trials in **Table S3**.

##### 5.1.1.3. Other DDR Agents

The ATR inhibitor Ceralasertib (AZD6738) is being investigated as monotherapy in chemotherapy-resistant TNBC as part of a pre-surgical window of opportunity and post-surgical biomarker study (NCT03740893, PHOENIX), reviewing the change in mean proliferation index between baseline and post-treatment. PARTNERING is a phase 2 sub-study for the PARTNER trial that offers durvalumab along with AZD6738 to patients with evidence of residual disease after completion of NACT and before surgery. WEE 1 inhibitors have not yet been reviewed in the early TNBC setting.

**Table S3** summarises the major incomplete clinical trials involving DDR agents in patients with stage I–III TNBC.

**Summary Box 4—DNA damage response: treatment strategies**

−There is strong evidence to support the addition of platinum agents to NACT to improve patient outcomes, especially in high-risk and g*BRCA* carriers.
−Improvements in pCR and EFS rates with platinum chemotherapy combinations need to be balanced against additive chemotherapy toxicities.
−PARP inhibition causes replication stress, induces ssDNA breaks and affects the normal regulation of p53 and its downstream effectors.
−Encouraging evidence supports the efficacy of single agent PARPi in BC patients with g*BRCA* mutations who have no prior chemotherapy exposure.
−The group of patients with TNBC most likely to benefit from PARP inhibition in the neoadjuvant setting is yet to be established.
−Olaparib improves DFS in g*BRCA* carriers with high-risk HER2 negative disease following neoadjuvant or adjuvant chemotherapy.

#### 5.1.2. Predictive Biomarkers of DDR Agents Response

##### 5.1.2.1. *BRCA* Mutations

The predictive value of both g*BRCA* and somatic *BRCA* mutations for response to platinum and PARPi has been validated in large clinical trials that included patients with ovarian and metastatic BC (108, 114). The role of *BRCA* status as an independent predictive biomarker for the TNBC population in the neoadjuvant setting is still unclear, with studies showing conflicting results. In a secondary analysis of the GeparSixto trial (*n = 50*) (119), *gBRCA* mutations were predictive of higher pCR rates and carboplatin did not increase this further. In the CALGB 40603 trial, pCR rates in patients with g*BRCA* mutations were similar to the overall population, and this outcome was not altered by the addition of carboplatin (120).

*BRCA1*/2 mutation carriers with the TNBC subtype in the I-SPY 2 trial were significantly more likely to achieve a pCR than non-*BRCA* TNBC (predicted pCR of 75% vs. 29%) (121) and a greater response was seen for patients with a *BRCA*-ness signature (116). Subgroup analysis of the BrighTNess trial did not show a difference in pCR rate based on *BRCA* status (93). However, in the GeparOcto trial (122) g*BRCA* mutation carriers gained greater benefit from platinum (68.1% vs 45.7%, p = 0.005), particularly in the TNBC subgroup (74.3% vs 47%, p = 0.005).

In the PETREMAC trial, in which patients received olaparib monotherapy before chemotherapy, pathogenic mutations (germline or somatic) in the HR pathways and/or *BRCA1* promoter methylation were associated with olaparib and its overall response (OR) of 88.9% (109). Although pCR rates in the GeparOLA trial for *gBRCA1*/2 carriers were significantly higher than those in non-carriers (62.7% vs 41.3%, P = 0.047), exploratory analysis revealed no difference between treatment arms if somatic or germline *BRCA1*/2 mutations were detected (110).

##### 5.1.2.2. HRD by Gene Set Analysis and Functional Assays

Several attempts to simplify and systematically identify common molecular changes associated with defective HR have been published. The evaluation of DNA damage repair-related genes by either gene expression or by the presence of mutations has shown a positive association with response. Confirmation of the predictive value of these individual efforts has not always been accomplished given the underlying heterogeneity of some of these variations (**Table 7** **)**.

**Table 7:** HRD related biomarkers and its association with treatment response.

Clinical cohort	Biomarker	Type of response association	Ref.
**Gene sets and functional assays**		
Prat et al. (2014) Five independent cohorts n = 1,055	Cell cycle-related genes (CCNE1, CHEK1, CCNB1, and FANCA)	High gene expression - increased response	(123)
	Endocrine response (*PGR, FOXA1, CCND1*, and *IL6*)	Low gene expression - increased response	
	EMT (*TWIST1* and *ZEB1)*	High gene expression - lack of response	
Severson et al. I-SPY trial n=116	77- gene signature ` BRCA-ness`	*BRCA*-ness signature positively associated with response (OR = 3.2, p = 0.03) in the Velaparib-carboplatin arm. Significant Biomarker * treatment interaction (p=0.025)	(116)
Graeser et al. (2010) n = 68	*RAD51* focus formation by immunofluorescence	Low *RAD51* score was strongly predictive of response (33% vs 3%, p = 0.01)	(124)
Eikesdal et al. (2021) n = 32		Low *RAD51* scores positively associated with Olaparib response.	(109)
Eikesdal et al. (2021) n = 32	*ATRX, BRCA1/2, EMSY*,	Mutations more frequent in responders (p = 0.011)	(109)
	*MSH6*, *PARP10, PPM1D)*		
	*PIK3CA*, *AKT1, KRAS, IGF2R*,	Mutations are more frequent in non-responders.	
	*NF2* and *TGFBR2*		
Peng et al. (2014) n = 295	230 gene HRD signature	Predictive of PARP inhibition sensitivity in cell lines.	(125)
		Association with overall survival in the BC patient cohort.	
**Genomic scars and mutational signatures**			
Vollebergh et al. (2014) n = 249	BRCA-like signature' by array comparative genomic hybridisation (aCGH) patterns	Better OS and PFS (HR 0.19, 95% CI: 0.08 to 0.48). No association with OR to carboplatin based - NACT	(126)
Eikesdal et al. (2020) PETREMAC trial n = 32	HRD by MLPA analysis of CNV	No significant association with response to Olaparib (p=0.07)	(109)
Telli et al. (2015) PrECOG 0105 trial n = 80	HRD -LOH and HRD-LST score	Mean HRD-LOH scores higher in responders vs nonresponders (P = .02) . Subgroup of BRCA1/2 germline mutations carriers excluded association remained significant (P = .021)	(127)
Loibl et al. (2018) GeparSixto trial n=588	HRD score >42: unweighted sum of HRD-LOH, HRD-TAI, HRD-LST and BRCA1/2 mutations	HRD high vs HRD low pCR (55.9% vs 29.8%, p=0.001). Greater pCR rates when HRD high tumours were treated with platinum (64.9% vs 45.2%, p=0.025). Patients with no gBRCA mutations: high HRD associated with higher pCR rate (49.4% vs 30.9%, p=0.050) irrespective of the use of carboplatin.	(128, 129)
Fasching et al. (2020) GeparOLA trial n = 107		In the paclitaxel Olaparib arm, a pCR rate of 55.1% (90% CI 44.5% to 65.3%)	(110)
Staaf et al. (2019) SCAN-B trial n = 144	HRDetect: : microhomology-mediated indels, HRD index,SBS3, RS3, and RS5	HRDetect-high associated with better DFS outcomes compared to HRDetect-low (HR 0.31, 95% CI = 0.13–0.76)	(130, 131)
Chopra et al. (2020) RIO trial n = 27		HRDetect score >0.7 not associated with Ki67 change after PARP inhibitor treatment.	

##### 5.1.2.3. HRD by Genomic Scars and Mutational Signatures

The detection of mutational signatures that uniquely identify patterns of defective HR repair has been the subject of several studies. Vollebergh et al. assessed whether array comparative genomic hybridisation patterns could predict the benefit of intensified carboplatin-based chemotherapy (126). An HRD score defined by an unweighted sum of loss of heterozygosity, telomeric allelic imbalance, large-scale transition, and *BRCA*1/2 mutations has been tested in TNBC treated with platinum, and used to aid patient selection in PARPi trials (128, 129). In the absence of g*BRCA* mutations, a high HRD score was associated with higher pCR rates irrespective of the use of carboplatin. The microhomology-mediated indels, HRD index, single base substitution signature 3, rearrangement signatures 3 and 5, and genomic instability markers of HRD are aggregated into the HRDetect score (91). The prognostic value of HRDetect has been demonstrated in two retrospective clinical cohorts, and further evaluation of its predictive power in randomised clinical trials is awaited.

HRD has yet to be used to guide the clinical management of TNBC despite its theoretical significance. The absence of a standardised definition of HRD beyond g*BRCA* mutation and the lack of prospective clinical trial data currently limits its clinical utility.

##### 5.1.2.4. Tumour Mutational Burden

More tumour mutations could be correlated with an enhanced response to drugs causing DNA damage. For example, somatic hypermutation was shown to be an independent factor for estimating the risk of platinum sensitivity in high-grade serous ovarian cancer (OR = 3.616, p = 0.002) (132). A higher tumour mutational burden (TMB) has been observed in BCs that harbour DDR gene mutations (133), although the correlation with response to platinum is not yet established. In the PETREMAC trial, no difference in TMB was observed between responders and non-responders, or *BRCA* carriers versus non-carriers (109).

**Summary Box 5—DNA damage response: Biomarkers**

−The role of *BRCA* status as an independent predictive biomarker among the TNBC population in the neoadjuvant setting is unclear.
−Overall, alterations in DNA damage repair-related genes by either gene expression or presence of mutations has shown a positive association with response to NACT and/or PARPi.
−Mutational signatures predictive of *BRCA1/BRCA*2 deficiency or a `*BRCA*-ness status` have shown a trend to positive association with response to platinum chemotherapy. However, these results are signature specific and should be considered preliminary. Data from randomised clinical trials that prospectively assess the value of these biomarkers is awaited.
−Higher TMB has been observed in BC tumours that harbour DDR gene mutations. Correlation with response to platinum agents is not yet established.

### 5.2. Immune Response

Although BC is largely considered an immune-quiescent cancer type (134), increasing evidence suggests that a range of tumour immunogenicity is present. TNBC is characterised by increased immune activation and wide immune heterogeneity compared to other BC subtypes (135).

#### 5.2.1. Therapeutic Approaches

Tumours evade detection and eradication by the immune system through the dysregulation of pathways controlled by immune checkpoints. Immunotherapy harnesses the immune system of the patient to target malignant cells using immune checkpoint inhibitors (ICI), chimeric antigen receptor T cells, or cancer vaccines. ICIs release the immune system from tumour-induced inhibitory signals, allowing an effective anti-tumour response. They include monoclonal antibodies (mAbs) against cytotoxic T lymphocyte-associated antigen-4 (CTLA-4), programmed cell death-1 (PD-1), and programmed cell death ligand-1 (PD-L1).

##### 5.2.1.1. Monoclonal Antibodies Against PD-1

Pembrolizumab is the most well-established and successful anti-PD-1 ICI in operable TNBC. The addition of pembrolizumab to NACT has shown increases in pCR rates across several RCTs, including the KEYNOTE-173 and I-SPY 2 trials (136, 137). These successes led to the landmark phase 3 KEYNOTE-522 trial, which has culminated in the FDA approval for use of pembrolizumab in high-risk early-stage TNBC, the first regulatory approval for an immunotherapy agent in this setting. Pembrolizumab is now considered a standard of care treatment in the United States for patients fitting trial eligibility criteria.

KEYNOTE-522 evaluated neoadjuvant pembrolizumab along with carboplatin/paclitaxel and anthracycline-based NACT, and then adjuvantly as monotherapy, in high-risk early TNBC. The pCR rate improved by 7.5% (95% CI: 1.6 to 13.4%) with the addition of pembrolizumab, and after a median follow-up of 39.1 months, 36-month EFS improved from 77 to 85% (HR: 0.63; 95% CI, 0.48 to 0.82; P <0.001). OS data remains immature at the time of analysis (34). High-risk patients derived the greatest benefit, with higher absolute improvements in pCR in stage III and node-positive disease. There are some limitations to this study. With this trial design, it is not possible to elucidate the relative contributions of the neoadjuvant and adjuvant treatment phases to these EFS results. Concern has been raised at the rate of serious adverse events (77% incidence of grade ≥3 events in the immunotherapy group) and immunotherapy-related adverse effects (irAE) (affecting 33.5% of patients on this trial) due to their protracted nature. It is therefore imperative to detect predictive biomarkers to facilitate the selection of patients likely to derive the most benefit from immunotherapy and treatment de-escalation strategies. No predictive biomarkers were identified in this trial. Improvement in pCR rate was seen regardless of PD-L1 status (138). Patients on the pembrolizumab arm that achieved pCR derived a modest survival benefit (approximately 2%), as compared to 10% in the cohort of patients with residual disease at surgery. This suggests that the value of adjuvant pembrolizumab as a monotherapy may be small in the group that achieved pCR. Removal of the adjuvant portion of treatment based on response to surgery could represent a potential treatment de-escalation strategy that requires further exploration.

##### 5.2.1.2. Monoclonal Antibodies Against PD-L1

Atezolizumab, durvalumab, and avelumab are the most established anti-PD-L1 ICIrs being investigated in operable TNBC, although results from trials have been inconsistent. The pCR rate improved from 41 to 58% with the addition of atezolizumab to anthracycline/taxane-based chemotherapy in Impassion031 (139). Secondary endpoints (EFS, DFS, and OS) are expected later this year. However, this trial is not powered to show survival differences. The phase 3 NeoTRIPaPDL1 trial failed to show a significant pCR advantage with the addition of atezolizumab to neoadjuvant carboplatin and nab-paclitaxel (140), although EFS was the primary endpoint and this data is not yet available. These incongruent results are likely to reflect the higher-risk patient population in NeoTRIPaPDL1 and the difference in the chemotherapy backbone. Results from the TONIC trial suggest anthracycline chemotherapy, used in Impassion031, leads to a potentiation of the effects of immunotherapy (141). These insights should inform the choice of chemotherapy backbone in the design of future immunotherapy trials.

GeparNUEVO assessed Durvalumab in addition to anthracycline/taxane-based NACT. This showed a non-significant 9% improvement in pCR rate. Improvements in 3-year iDFS and 3-year OS were also seen, though this trial was not powered to definitively assess long-term survival differences. An underpowered subgroup analysis showed a particular benefit in patients who received durvalumab alone for two weeks prior to NACT, suggesting immunological interactions with priming in this window phase (142, 143). While the small patient cohort included in GeparNUEVO has resulted in statistically non-significant pCR and iDFS benefits, the results are similar to those from KEYNOTE-522. This is despite lacking a platinum agent and an adjuvant treatment phase. These represent potential treatment de-escalation avenues that could benefit from further exploration. Discrepancy between the magnitud of benefit for pCR rate and survival seen across both trials suggest the value of pCR as a marker for long term survival in immunotherapy trials requires further exploration. Published and ongoing trials of ICI have been summarised in **Tables 8**, **9**.

**Table 8:** Major neoadjuvant trials that include immune checkpoint inhibitors in patients with stage I–III TNBC.

Neoadjuvant Trials							
Trial	Phase	Disease Setting	Sample size (TNBC where available)	Treatment	Primary	Results	Ref.
					endpoint		
**Pembrolizumab**							
KEYNOTE-522	3	Neoadjuvant/adjuvant treatment of stage II or III TNBC	1174	Pembrolizumab vs. placebo in combination with paclitaxel and carboplatin, and followed by AC/EC chemotherapy. Patients also underwent adjuvant treatment with pembrolizumab or placebo	pCR	pCR 63 vs 56%	
					EFS	3y EFS 84.5 % vs 76.8% (95% CI = 72.2–80.7%)	(34, 138)
ISPY-2	2	Neoadjuvant treatment of high-risk stage II to III HER2 negative breast cancer	114	Paclitaxel +/- pembrolizumab followed by adjuvant doxorubicin + cyclophosphamide (AC)	pCR	pCR 60% vs 22%	
						EFS in patients with pCR 93%	(136)
						EFS in patients without pCR 70%	
KEYNOTE-173	1b	Neoadjuvant treatment of high-risk, early-stage TNBC	60 (10 per cohort)	Pembrolizumab in combination with a taxane with or without carboplatin, followed by doxorubicin and cyclophosphamide.	Safety and RP2D	Overall pCR 60%	(137)
				6 regimens were evaluated.		1y EFS in patients with pCR 100% 1yr EFS in patients without pCR 88%	
						1y OS 80–100%	
NeoPACT	2	Neoadjuvant treatment of stage I–III TNBC	121	Carboplatin & docetaxel plus pembrolizumab	pCR	Pending	
NeoImmunoboost	2	Neoadjuvant treatment of non-metastatic TNBC	53	Pembrolizumab in combination with nab-paclitaxel followed by EC	pCR	Pending	
PELICAN-IPC 2015-016	2	Neoadjuvant treatment of non-metastastic HER2- BC	81	Pembrolizumab in combination with EC-paclitaxel	pCR	Pending	
**Atezolizumab**							
NCT02883062	2	Neoadjuvant treatment of stage II – III TNBC	72	Carboplatin and paclitaxel +/- atezolizumab followed by adjuvant AC	TIL %	pCR 55.6% vs 18.8%	(144)
						p-value 0.018	
					pCR		
Impassion031	3	Neoadjuvant treatment of stage II–III TNBC	333	Atezolizumab vs placebo in combination with nab-paclitaxel followed by AC	pCR	pCR 58% vs 41% (P = 0.004)	(139)
						PD-L1-positive cohort 69% (95% CI: 57–79)	
					PD-L1 status		
NeoTRIPaPDL1	3	Neoadjuvant treatment of early, high-risk, locally advanced TNBC	278	Carboplatin and nab-paclitaxel +/- atezolizumab	EFS	pCR 43.5% vs 40.8% (p = 0.066)	(140)
NCT02530489	2	Neoadjuvant treatment of stage I-III operable TNBC who were non-responders to initial anthracycline and Cyclophosphamide chemotherapy	37	Atezolizumab and nab-paclitaxel	pCR	pCR 30%( 95% CI: 16-49%)	(145)
						(Historical controls 5%)	
GeparDouze	3	Neoadjuvant/adjuvant treatment of high-risk TNBC	1520	Neoadjuvant Atezolizumab vs. placebo in combination with paclitaxel and carboplatin followed by AC. 6 months of adjuvant atezolizumab or placebo.	EFS	Pending	
					pCR		
**Durvalumab**							
GeparNUEVO	2	Neoadjuvant treatment of early TNBC	174	Durvalumab vs placebo in addition to anthracycline/taxane based neoadjuvant chemotherapy	pCR	pCR 53.4% vs 44.2% p 0.287	
						3y iDFS 84.9 vs 76.9 (HR 0.48, 95% CI 0.24–0.97)	(142, 143)
						3y DDFS 91.4 vs 79.5 (HR 0.31, 95% CI 0.13–0.74)	
						3y OS 95.1 vs. 83.1 (HR 0.24, 95% CI 0.08–0.72)	
B-IMMUNE	1b/2	Neoadjuvant treatment of HER2 negative and TNBC	57	Durvalumab in addition to paclitaxel followed by ddEC	SAEs	Pending	
					pCR		
NCT02489448	1/2	Neoadjuvant treatment of stage I–III TNBC	69	Durvalumab in addition to nab-paclitaxel followed by ddAC	pCR	Overall pCR 44% (95% CI: 30–57%) PD-L1 positive subgroup 55% (95% CI: 0.38–0.71) PD-L1 negative subgroup 32% (95% CI: 0.12–0.56)	(146)

**Table 9:** Major adjuvant trials that include immune checkpoint inhibitors in patients with stage I–III TNBC.

Adjuvant Trials						
Trial	Phase	Disease Setting	Estimated Sample size	Treatment	Primary	Results
					endpoint	
IMpassion030	3	Adjuvant treatment of stage II–III TNBC	2300	Atezolizumab vs. placebo in combination with adjuvant anthracycline/taxane-based chemotherapy	iDFS	
						Pending
SWOG S1418/ NRG BR-006	3	Adjuvant treatment of stage II–III TNBC with residual disease or postive lymph nodes following NACT	1155	1 year of pembrolizumab vs. observation	iDFS	Pending
A-Brave	3	Adjuvant treatment of high risk TNBC following NACT	474	1-year avelumab vs. observation	DFS	Pending
MIRINAE	2	Adjuvant treatment of TNBC with residual disease following NACT	284	Atezolizumab with capecitabine vs capecitabine alone	5y iDFS	Pending

Ongoing trials evaluating PARP inhibitors in combination with immunotherapy can be found in **Supplementary Table 3**.

The use of ICIs in TNBC is an area of active research, although it is at an early stage, and long-term outcome data remain immature for the majority of the neoadjuvant trials. Concern regarding the use of pCR as a primary endpoint upon which to grant regulatory approval for neoadjuvant pembrolizumab was cited by the FDA, and long-term survival data is of particular interest (147). There is a paucity of data available to guide use of pembrolizumab in the adjuvant or post-neoadjuvant setting, particularly in combination with agents such as capecitabine or olaparib, used in more contemporary practice. This represents a challenge when adopting pembrolizumab as the standard of care treatment, and the results of trials investigating these issues are highly anticipated.

##### 5.2.1.3. Cancer Vaccines

Cancer vaccines use tumour associated antigens to stimulate CD4+ and CD8+ T cells, inducing the immune system of the patient to target cancer cells that were previously successfully evading immune suppression. They have yet to show success in late-stage clinical trials or to receive regulatory approval for TNBC. Clinical trials evaluating cancer vaccines in non-metastatic TNBCs are listed in **Table S4**.

**Summary Box 6—Immune response: Treatment strategies**

−Immunotherapy is of particular interest in TNBC due to the higher degree of immune activation seen in comparison to other BC types.
−TNBC is a heterogeneous disease that exhibits various degrees of immunogenicity.
−Several early stage BC trials have established PD-1 and PD-L1 ICIs as a promising treatment option in combination with chemotherapy.
−Pembrolizumab has been granted FDA approval in the neoadjuvant setting for high-risk early-stage TNBC in combination with chemotherapy and to continue as monotherapy in the adjuvant setting (KEYNOTE-522).

#### 5.2.2. Predictive Biomarkers of ICI Response

##### 5.2.2.1. PD-L1

PD-L1 expression is higher in TNBCs compared with non-TNBCs (135) and quantification is currently performed using five distinct FDA-approved companion diagnostic tests across tumour types. The variety in assays, scoring systems, and cut-off values renders the interpretation of its predictive value challenging (148). Increased pCR rate in PD-L1+ early-stage TNBC is seen, but rather confusingly, ICI benefit independent of PD-L1 status has been consistently described (138, 139, 143). In the GeparNUEVO trial, pCR rate was increased in PD-L1+ tumours in all therapy groups, but PD-L1 did not predict ICI response (143). Similar results were observed in the KEYNOTE-522 and Impassion 031 trials.

##### 5.2.2.2. Tumour Mutational Burden

High tumour mutational burden precipitates enhanced immunogenicity by increasing the number of tumour antigenic peptides or neoantigens that can be recognised by T cells (149). Based on this hypothesis, high TMB has been correlated with an increased response to ICI (150, 151) independently of PD-L1 expression (152). The FDA granted accelerated approval of pembrolizumab as monotherapy for advanced tumours that exhibit high TMB (defined as ≥10 mut/Mb) in 2020 (153). More recently, it has been shown that the association of TMB with response to ICI relies on a positive correlation between CD8+ T-cell level and neoantigen load and differs across tumour types (154).

Due to limited data availability and differences among TMB quantification methods, the role of mutational load as an independent predictive biomarker of ICI response is yet to be defined in TNBC. In the GeparNUEVO trial, TMB was higher in patients with pCR (median 1.87 versus 1.39 mut/MB), and both continuous TMB and the immune GE profile independently predicted pCR (155). In comparison, no difference in pCR rate was observed in patients with high TMB who received ICI compared with other targeted therapies in the ARTEMIS trial (NCT02276443) (156).

##### 5.2.2.3. Tumour Infiltrating Lymphocytes (TILs)

Both intra-tumoural TILs (iTILS) and stromal TILs (sTILs) have prognostic and predictive roles for treating early TNBC and have also been evaluated in this setting as biomarkers of immunotherapy response. In the GeparNUEVO trial (142), sTILs before therapy predicted a higher pCR rate overall and in both therapy groups, but were not predictive of durvalumab response. The increase in iTILs in post-window samples compared with pre-therapeutic samples was predictive of pCR, yet the treatment interaction test did not reach significance (P = 0.085). High TILs were significantly associated with the olaparib response in the PETREMACT trial (109). Criscitiello et al. used the LASSO penalised regression model to develop a 4-gene signature to predict high and low TILs after NACT. A high TIL signature was associated with improved long-term outcomes independent of pCR (157). Overall, increased TILs are associated with a more favourable response to NACT and improved long-term outcomes (158, 159).

##### 5.2.2.4. Immune Signatures

GE immune signatures have been extensively used to describe profiles of immune infiltration and immune cell types that impact on the prognosis of many tumour types, including TNBC (160–163). Few studies have tested the value of GE immune signatures in the prediction of chemotherapy response in the early setting of TNBC. In the SWOG 9313 trial, Sharma et al. (164) evaluated the performance of a DNA damage immune response signature and sTILs as prognostic markers in patients with TNBC treated with adjuvant doxorubicin and cyclophosphamide. DDIR was associated with improved OS and DFS and was moderately correlated with sTILs density (≥20% v, <20%). Using network analysis, Lv et al. identified CXCL9 and CXCL13 as prognostic biomarkers in TNBC. Further testing in two neoadjuvant data sets confirmed its predictive value in the response to chemotherapy (165). An exploratory analysis of the GeparNUEVO trial revealed that predefined TIL and IFN-gamma signatures were associated with an increased pCR rate, without specificity for durvalumab response. The expression of six genes required for immune cell function was significantly correlated with pCR and showed a positive test for interaction with durvalumab plus NACT (166). Further evaluation of the interactions between the tumour and the immune system, as well as its architectural heterogeneity, will provide a more accurate estimation of the individual predictive potential to be derived from immune signatures.

##### 5.2.2.5. Microsatellite Instability Status

Pembrolizumab monotherapy received FDA approval in 2017 for treating advanced mismatch repair deficient solid tumours (167). Although only a small proportion of breast cancers are defined as microsatellite instable, (168) tumours with defects in the mismatch repair pathways are known to have highly upregulated expression of multiple immune checkpoints and increased sensitivity to ICI (169). The introduction of new strategies to facilitate the identification of this biomarker in a low-frequency cohort like TNBC remains a challenge.

**Summary Box 7—Immune response: Biomarkers**

–Response to ICIs appears to be independent of PD-L1 status in early TNBC.
–High TMB has been correlated with an increased likelihood of response to ICI, particularly in tumours where CD8+ T-cell levels are positively correlated with neoantigen load.
–The role of mutational load as an independent predictive biomarker of ICI response is yet to be defined in TNBC.
–Increased TILs are associated with a more favourable response to NACT and long-term outcomes.
–Modest positive association of GE immune signatures with ICI response has been reported.
–The interaction between TMB and GE immune signatures has been shown to be a promising independent predictor of pCR.
–The dynamics of immune activation after treatment are strongly associated with long term outcome, independently of response rate.
–Tumours with defects in the mismatch repair pathways are known to have highly upregulated expression of multiple immune checkpoints and increased sensitivity to ICI.

### 5.3. PIK3CA/AKT1/PTEN Pathway

Dysregulation of the PI3K/AKT/mTOR pathway is often observed in TNBC (24, 32), and remains a promising target for the future treatment of this BC subtype. Pathway activation is predominantly *via PIK3CA* mutations (~9–18%), loss of *PTEN* (~35%), or *INPP4B* (~30%), and amplification of *PIK3CA (~43%)*. The frequency of PI3K/AKT/mTOR pathway activation and its spectrum varies by TNBC subtype (24, 32), and is strongly associated with the LAR subtype across classifiers.

#### 5.3.1. Alpha-Specific PI3K Inhibitors

In unselected TNBC, response to PIK3CA inhibitors remains low. The BELLE-4 study evaluated the efficacy of buparlisib in the locally advanced setting for patients with HER2-negative BC along with paclitaxel versus placebo and observed no benefit from PIK3CA inhibition. Worse outcomes were observed in the TNBC cohort treated with the PIK3CA inhibitor, and the lack of benefit was independent of *PIK3CA* mutation or PTEN loss by immunohistochemistry (170). Shorter treatment duration in the buparlisib arm due to adverse events and longer progression-free survival (PFS) in the placebo arm than anticipated are possible explanations for the worse outcomes in this subgroup. The global lack of activity is possibly due to inadequate patient selection and the absence of an accurate biomarker. Parallel pathway activation could also explain a resistance mechanism that requires addressing.

#### 5.3.2. AKT Inhibitors

Ipatasertib was reviewed in the neoadjuvant setting along with paclitaxel for TNBC patients in the FAIRLANE trial (171). Adding ipatasertib did not significantly increase the pCR rate compared with paclitaxel alone, and this effect was independent of *PIK3CA/AKT1/PTEN* or *PTEN* low status. A complete clinical response was absent in the placebo-treated group in patients with tumours defined as the LAR subtype, but was observed in 50% of those treated with ipatasertib. This difference was not evident in pCR rates. Elevated immune scores were more strongly associated with improved outcomes in paclitaxel-treated compared with ipatasertib-treated patients, highlighting the key interaction with the immune system. All ipatasertib-treated patients with low immune scores and complete clinical response had *PIK3CA/AKT1/PTEN-*altered tumours. MK2206 has been trialled in the neoadjuvant setting in the I-SPY2 trial for stage 2–3 BC of any subtype (172). Patients received paclitaxel chemotherapy with or without MK2206, then AC. pCR for the TNBC group was 40.2% with MK2206 vs. 22.4% without. Following assessment of biomarkers in the AKT pathway in the TNBC subgroup, higher levels of phosphorylated AKT and its substrates were paradoxically associated with a reduced response to MK-2206.

#### 5.3.3. MTOR Inhibitors

Everolimus has been reviewed in the neoadjuvant setting for patients with TNBC along with cisplatin and paclitaxel (173), and along with docetaxel, 5-fluorouracil, epirubicin, and cyclophosphamide (174). No improvement in the response rate has been demonstrated.

The exact contribution of drugs targeting the PIK3CA/AKT1/PTEN pathway in early TNBC has not yet been defined. The complexity of the immune microenvironment and parallel molecular alterations can obscure accurate estimation of clinical benefit if they are not in some way accounted for. It is important to try these therapies in a way that reduces these confounders and separates the TNBC subtypes to determine their individual responses. Current approaches include combining alpelisib with nab-paclitaxel in the neoadjuvant setting (NCT04216472) for anthracycline refractory TNBC with PIK3CA or PTEN alterations, with exploratory objectives to assess biomarkers of response and resistance to alpelisib and nab-paclitaxel combination.

**Table S5** summarises ongoing trials that target this pathway in early TNBC.

### 5.4. AR Pathway

AR expression is found in approximately 10–35% of TNBCs as detected by immunohistochemistry (175, 176). The LAR molecular subtype derived from GE accounts for 20–40% of TNBC and is characterised by the activation of AR, ER, prolactin, and ErbB4 signalling. Tumours defined as the LAR subtype typically contain a higher number of PIK3CA mutations, and the pCR rate following NACT is significantly lower compared to other subtypes. (18–20).

There is a paucity of data for drugs targeting the AR pathway in the early TNBC setting. Enzalutamide has been trialled as monotherapy (177), and along with PIK3CAi in the advanced setting with modest benefit (178). Other AR pathway targeted drugs, for example, abiraterone and bicalutamide, have been reviewed in the advanced setting with modest results (179, 180). Although the overall benefit remains limited, it is unclear if this derives from inadequate patient selection or analogous pathway activation. Results from four trials in the early TNBC setting are highly anticipated.

**Table S5** summarises ongoing trials that target this pathway in early TNBC.

### 5.5. Receptor Tyrosine Kinase Family

#### 5.5.1. HER2

Approximately 35% of TNBCs as defined by immunohistochemistry could be classified as HER2-low (181). Somatic ERBB2 mutations occur in approximately 3% of TNBC (6), and a subset of TNBC tumours are classified as HER2-enriched by gene expression. This biological heterogeneity has expanded therapeutic opportunities in this population of patients. In an exploratory analysis of a cohort of the I-SPY2 trial, activation of HER2-EGFR was identified as a positive predictor of pCR in 49 TNBC patients treated with a pan-HER inhibitor (182). A significant correlation between the response to HER2 inhibition and HER2 pathway activation has been demonstrated in TNBC cell lines (183).

Neratinib has been investigated in the neoadjuvant setting for high-risk clinical stage II or III BC. The pCR rate overall in the I-SPY 2 trial was 37.5% in the neratinib arm, and among patients demonstrating the phosphorylation of *HER2* or *EGFR* (i.e., biomarker-positive for *EGFR* Y1173 or *ERBB2* Y1248), it rose to 63% (184). Encouraging results in the HER2-low–expressing refractory BC setting with Trastuzumab Deruxtecan *(*OR 37%) (185) and Trastuzumab Duocarmazine (OR 40%) (186) now require translation into the early setting. These trials illustrate the importance of identifying patients categorised as TNBC who are more accurately defined as HER2 low **(** **Table S5** **).**

#### 5.5.2. VEGF

*VEGF* promotes angiogenesis, invasion, and increases vascular permeability and is an essential element in TNBC formation, progression, and metastasis. VEGF-A expression is higher in TNBC compared with other BC subtypes (187), and enhanced angiogenic potential is associated with poor prognosis in BC (188). Targeting of *VEGF* has been extensively tested in TNBC, but no clear predictive biomarkers of treatment response have been identified.

Trials targeting VEGF in the neoadjuvant TNBC setting have shown disappointing results to date, with no difference in DFS or OS. The addition of bevacizumab significantly increased the rate of pCR among patients with Her2-negative disease in some studies (103, 189–191). The ARTemis and GeparQuinto trials reported increased benefits primarily in the TNBC subgroup. In the adjuvant setting, the BEATRICE trial added bevacizumab to anthracycline and/or taxane-based chemotherapy (192), and no difference in iDFS or OS between treatment groups was found. The underlying reason for the lack of treatment effects with these drugs is poorly understood. It is possible that a fundamental flaw in either the drug or the signalling pathway is being overlooked. Attempts to overcome drug resistance using novel agents and combinations are ongoing (**Table S5**).

#### 5.5.3. FGFR

The fibroblast growth factor receptor family includes *FGFR1–4*. Signalling through this pathway regulates cell survival, proliferation, and differentiation. Genes that encode for these receptors are amplified in ~10% of BC (24). Although *FGFR1* is the most frequent genomic alteration in all subtypes of BC, amplification and overexpression of FGFR2 are more frequently observed among TNBCs (~4%). Basal BC with elevated *MET* and *FGFR1* signatures is associated with poor relapse-free survival (193). The interplay between MET and FGFR regulates cancer stem cells in mesenchymal subtypes (194).

Trial data in this setting is limited to a few studies that do not select for TNBC but in which some response to this target has been seen. It seems likely that the correct biomarker has not yet been identified. Ongoing trials for this target in the neoadjuvant setting include a window of opportunity trial combining lenvatinib and pembrolizumab (NCT04427293).

#### 5.5.4. EGFR

*EGFR* dysregulation is frequently reported in TNBC (195) and enrichment for this pathway signalling is predominantly observed in BL2 tumours (196). In contrast to EGFR mutations, EGFR amplification is a relatively frequent event (11% vs 23%, respectively) (24, 197) and is considered an independent prognostic factor for poor disease-free survival (198). Several attempts to target this pathway with tyrosine kinase inhibitors and mAbs in the context of mTNBC have been pursued without success. A limited number of trials have used these therapies in the early setting.

Trials are underway in the locallyCetuximab has been trialled along with neoadjuvant docetaxel in a pilot phase two study, including stage II–IIIA TNBC (199). The pCR rate was 24% [95% CI: 7.3–40.7] and the pre-therapy ratio between CD8+ and FOXP3+ TILs equal or higher than 2.75 was predictive of pCR (43% versus 0%). In addition, panitumumab and the EGFR/HER2 inhibitor lapatinib failed to demonstrate additional benefit in the advanced setting independent of EGFR activation (200, 201). The paucity of accurate biomarkers predictive of sensitive patients has led to unsatisfactory outcomes and limited clinical utility despite increasing evidence for EGFR as the driver of tumorigenesis in some TNBC.

**Table S5** summarises ongoing trials that target these pathways in early TNBC.

### 5.6. Other Oncogenic Targets

Inter-chromosomal rearrangements causing *NTRK* gene fusions can result in constitutive activation of TRK proteins, which act as oncogenic drivers through activation of cellular growth pathways. Results from early phase trials that included advanced NTRK fusion-positive solid tumours support the use of larotrectinib and entrectinib in this subgroup (202, 203). *NTRK* gene fusions occur at low frequency (~0.3%) among all solid tumours (203). However, a high prevalence is observed in a subgroup of TNBC (16). The *ETV6-NTRK3* gene fusion is frequently found in human secretory breast carcinoma (16), and although the vast majority of these breast tumours are treated with local treatments, targeting TRK signalling remains an option for cases of locally advanced disease.

*Trop-2/TACSTD2* is a calcium signal transducer with extracellular, transmembrane, and intracellular domains, and is overexpressed in many epithelial cancers, including TNBC. It stimulates cancer cell growth and is implicated in various metabolic pathways. TROP-2 has also been found in stem cells of various tissues, particularly in basal cells (204). Sacituzumab govitecan, a humanised mAb that targets TROP2, has shown a PFS and OS benefit in mTNBC (205). Trials are upcoming in the neoadjuvant setting (NeoSTAR, NCT04230109), and recruiting in the adjuvant setting (GBG102-SASCIA NCT04595565 as monotherapy and ASPRIA NCT04434040 along with immunotherapy) for patients with residual invasive disease after NACT.

Dysregulation of the *NOTCH* pathway leads to aberrant self-renewal and transformation of mammary cancer stem cells, resulting in tumourigenesis (206). Inhibition of *NOTCH* signalling is an attractive strategy for treating TNBC given its role in promoting EMT and cancer stem cell maintenance (207). Preclinical and clinical studies involving γ-secretase inhibitors and mAbs against *NOTCH* receptors have explored its potential utility with encouraging results, but toxicity has been limiting (208). The subgroup of TNBCs achieving the best response to the targeting of this pathway remains undefined.

Activation of *RAS/MAPK* signalling is more frequent in TNBCs compared to other BC subtypes, and it is typically associated with shorter survival (209, 210). Although canonical aberrations in the RAS, RAF, MEK, or ERK genes are not found frequently in TNBC, amplification or mutations in these genes are described in approximately 6% of BC overall (24). Other mechanisms for RAS/MAK activation have also been described (211). MEK inhibitors have been trialled in unselected mTNBCs with modest results (212, 213). Trials are underway in the locally advanced setting that select for hyperactivation of ERK (NCT04494958) and RAS pathway mutations (NCT05111561).

Dysregulation of the *JAK/STAT3*, *cyclinD–CDK4/6–INK4–Rb–E2F*, *TGF-β*, and *WNT/B-catenin* pathways appears critical in TNBC development and progression. Clinical testing of the inhibition of these pathways in TNBC is still immature.

**Table S5** summarises ongoing trials that target the above pathways in early TNBC.

**Summary Box 8—Other pathways: Treatment strategies**

–Targeted therapies should be guided by a biomarker to best determine efficacy in the TNBC population most likely to derive benefit.
–Dysregulation of the *PI3K/AKT/mTOR* pathway is often observed in TNBC. Efforts to target this pathway have inconsistently shown a modest benefit.
–Targeting AR has shown some clinical benefit, and several trials are ongoing to further evaluate this. A standardised method to determine AR pathway activation is lacking.
–Overall benefit of targeting the *EGFR, VEGF*, and *FGFR* pathways remains modest. The lack of predictive biomarkers that identify sensitive patients has limited the clinical utility of these drugs.
–Treatment directed towards HER2-low TNBC has provided new therapeutic opportunities in a proportion of patients, with encouraging results from trials to date.
–Sacituzumab govitecan, a humanised mAb that targets TROP2, has shown a PFS and OS benefit in mTNBC. It remains to be seen if this success can be translated into the early TNBC setting.

## Discussion

**Figure 1:**
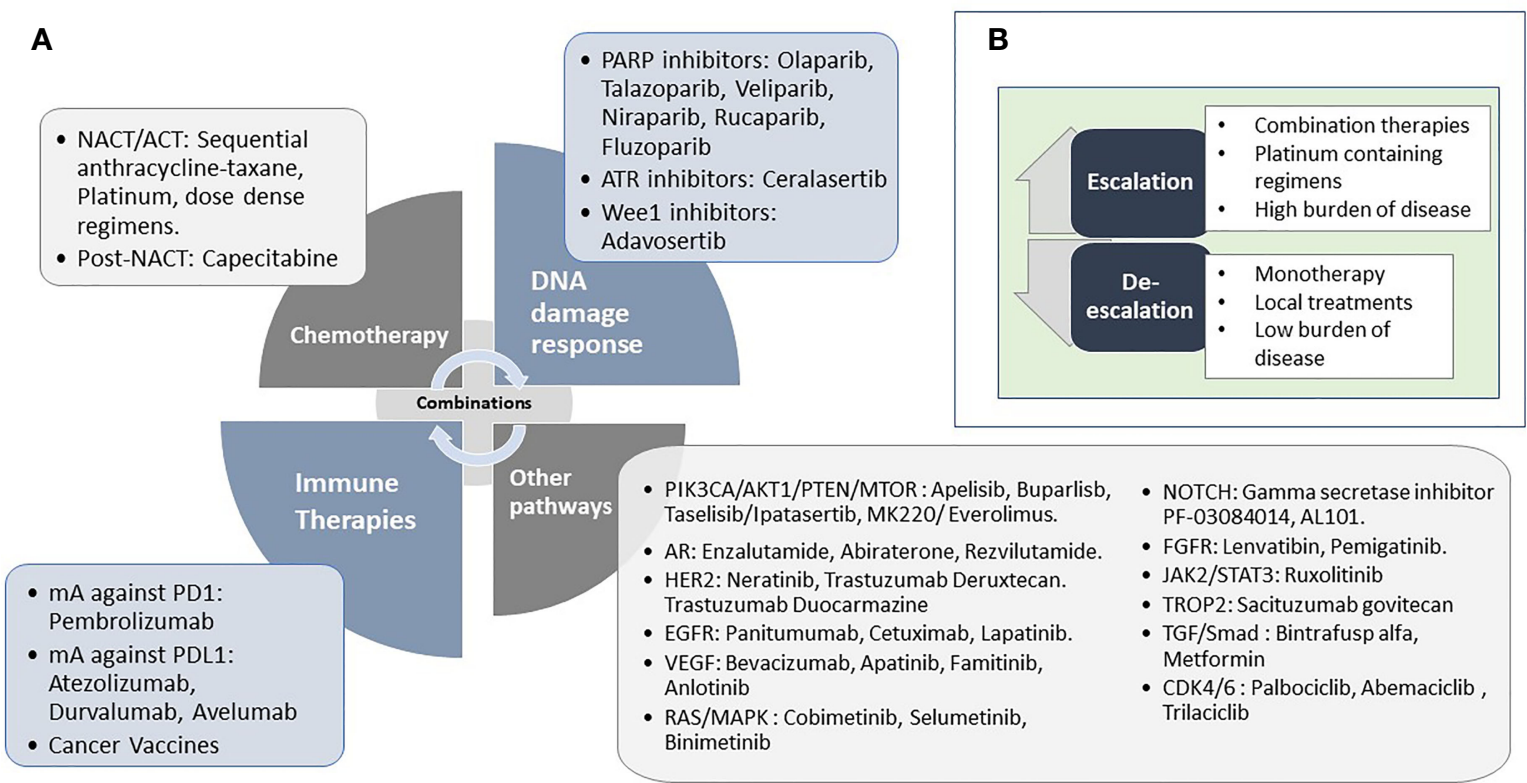
Current therapeutics strategies in early TNBC. **(A)**. Treatment spectrum **(B)**. Treatment modalities for escalation and de-escalation.

Improved understanding of tumour genomics, transcriptomics, epigenetics, and their interaction with the tumour microenvironment has allowed a greater insight into the true diversity of TNBCs. Additionally, numerous advances in both preclinical and clinical research have directed the treatment of TNBC towards a more personalised approach. Despite the introduction of an increasing number of novel strategies in the clinical setting, approximately one-third of patients diagnosed with early stage disease will have limited response to primary treatment and face a poor long-term outcome. The underlying complexity of TNBC and the challenges in translating experimental science into the clinic could explain why current management approaches remain insufficient. The current therapeutic landscape for early TNBC is severely limited compared to the large number of compounds in development. **Figure 1** shows the spectrum of agents with known or potential activity in TNBC. Only a small proportion of these reach patient care, and the pace at which these agents enter the early BC setting remains frustratingly slow. Immunotherapy and DDR agents lead the field with encouraging results.

Predictive biomarkers are not routinely used in the clinical management of early sporadic TNBC. The use of *gBRCA* mutations to select patients who could benefit from platinum-based chemotherapy and PARPi demonstrates how a molecular alteration can aid patient selection for treatment. As yet, there is no definitive evidence to either support or refute the use of PARPi in the non-g*BRCA* TNBC population. An ongoing neoadjuvant study (NCT03150576) that includes both sporadic TNBC and *BRCA*-associated tumours will help elucidate the value of *gBRCA* mutations in predicting the response to the addition of PARPi to platinum-based chemotherapy (100). Furthermore, no biomarker was predicted for benefit from Pembrolizumab in the KEYNOTE-522 trial, despite the encouraging response rates shown. The expected role of PD-L1 as a biomarker of response has not been proven in the early setting (138, 139). Substantial differences between the clonal architecture and the microenvironment of primary and metastatic tumours (214, 215) suggest that the role of a given biomarker should be evaluated separately in both early and advanced settings.

**Figure 2:**
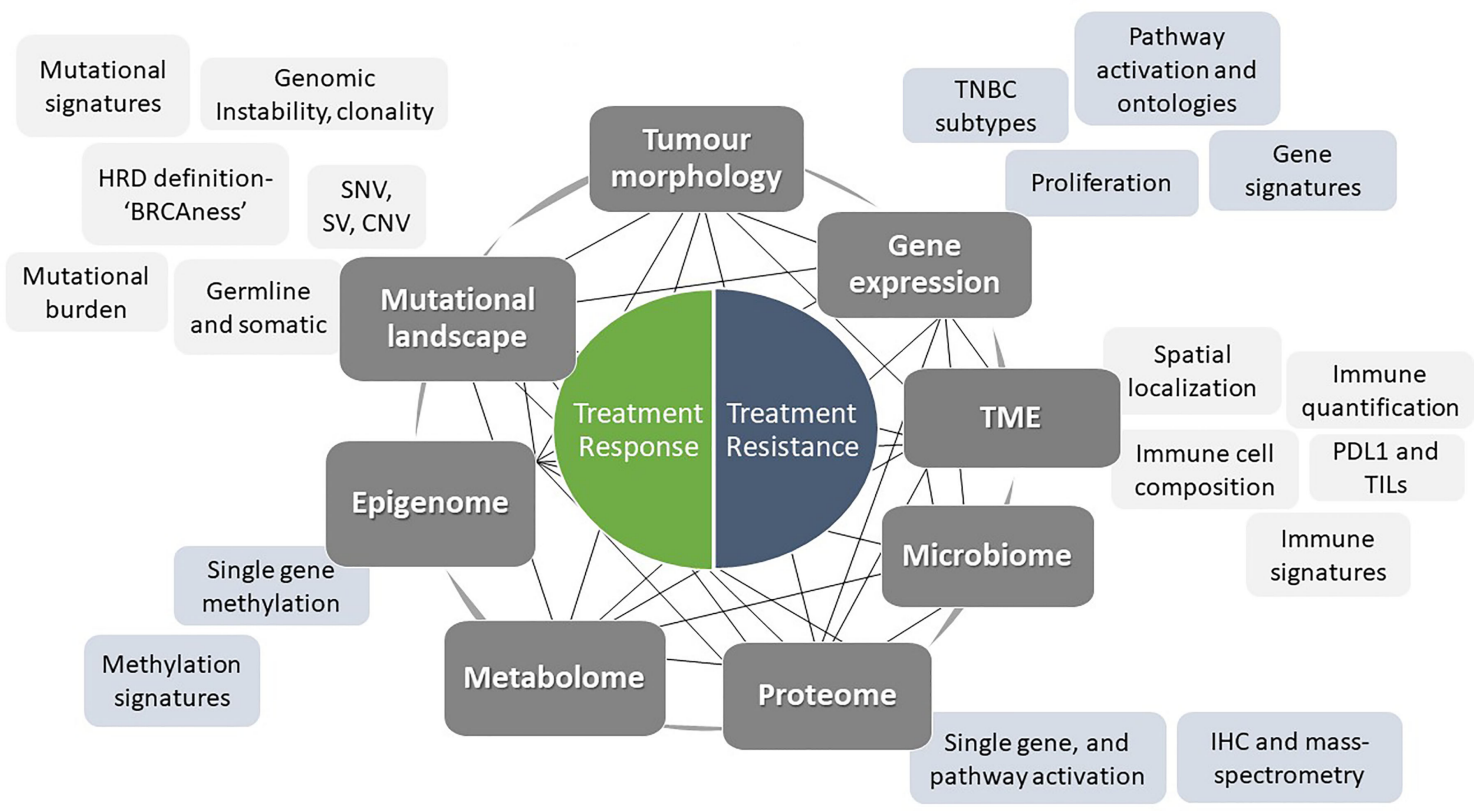
Biomarker landscape in TNBC.

**Figure 3:**
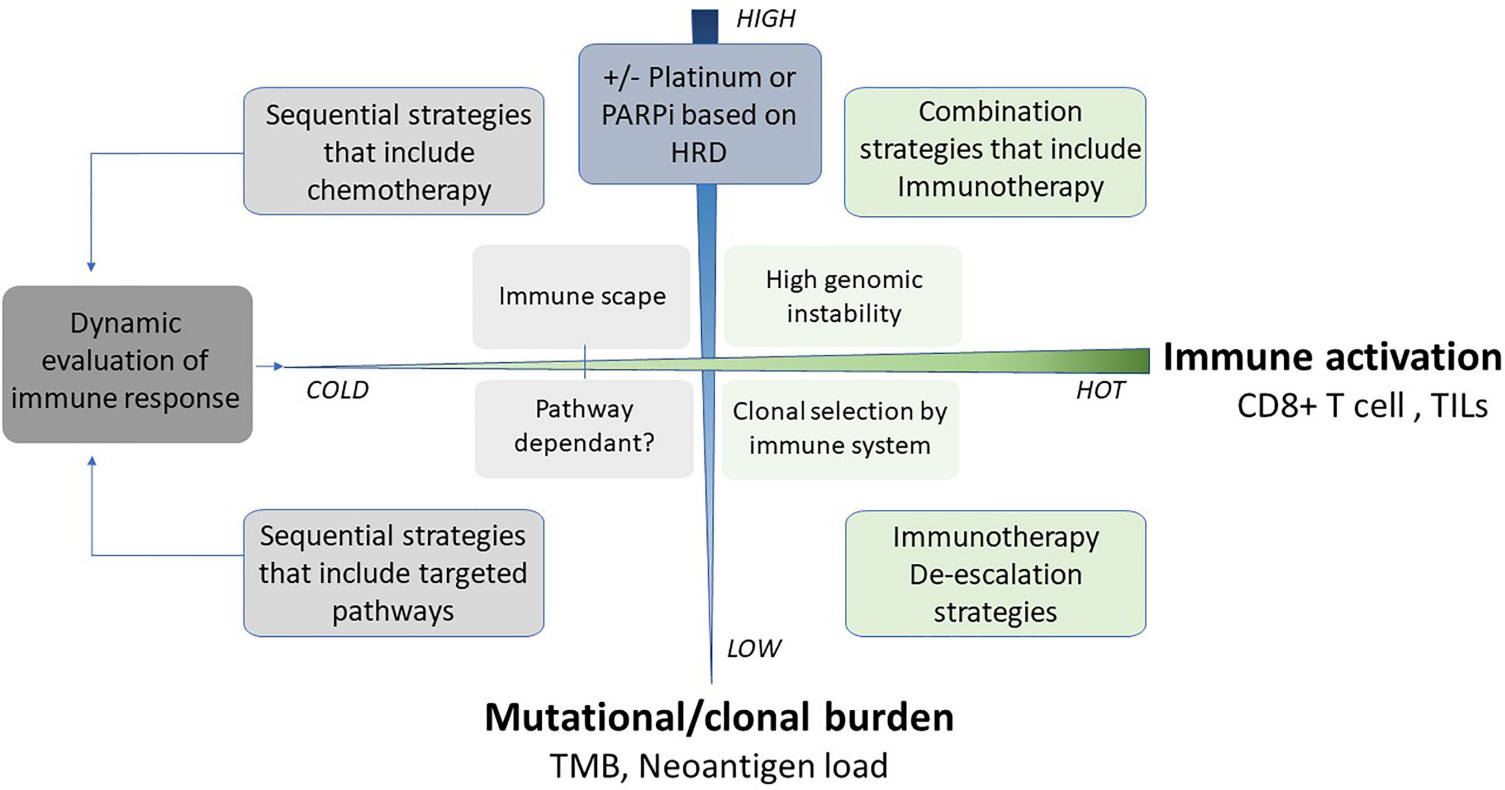
Proposed framework for the personalised treatment of early. TNBC.

A single biomarker strategy is unlikely to be successful for such a heterogeneous disease, considering the large number of treatment strategies already tested and the increasing evidence of molecular complexity in TBNC. **Figure 2** illustrates the variety of molecular components currently being explored as potential biomarkers of response and resistance. Several interactions across components also contribute to the challenge. As an example, to adequately characterise the relationship between host immunity and tumour, a single determination of the extent of immune activation is expected to be insufficient. Understanding how the immune response modulates the intrinsic genomic architecture of the tumour and the spatial and cellular distribution of immune cells in response to treatment appears to be crucial. Similarly, multiple pathway signalling, a common finding in TNBC tumours, could result in the activation of compensatory feedback loops that explain some mechanisms of tumour evasion and resistance when a single pathway inhibition is applied (216). An integrative approach including tumour architecture, microenvironment, and pathway activation is more likely to succeed. A pragmatic example of how an immune-molecular profile directed approach could be implemented is shown in **Figure 3**. Tumours could be classed as ‘hot’ (high immune activation) or ‘cold’ (low immune activation) as well as ‘high-burden’ (high mutational/clonal burden) or ‘low-burden’ (low mutational/clonal burden). Hot-high burden tumours are frequently highly proliferative and more likely to exhibit high chromosomal instability. Increased response to cytotoxic and immunotherapy agents is anticipated in this subgroup. The hot-low burden group represents a subgroup in which clonal selection has been enforced by an active immune system. This good-prognosis subgroup is likely to require less intensive therapies with treatments focused on targeting key drivers. In sharp contrast, cold tumours require more comprehensive approaches that often include treatment escalation strategies. It is possible that due to quiescent mechanisms of tumourigenesis, cold tumours remain invisible to the immune system. Therefore, sequential strategies that aim to enhance the effect of the immune system are essential in this group. In cold-low burden tumours, targeted pathway inactivation followed by immune checkpoint inhibition could potentially result in an augmented immune response, achieving long-lasting control of the disease. Cold-high burden tumours constitute a poor prognosis group with patent mechanisms of immune evasion. Sequential strategies that include immunotherapy followed by either chemotherapy, pathway-specific targeted agents, or radiotherapy-targeted agent combinations are plausible options.

Response to NACT, measured as the amount of residual disease found at surgery, has recently been used as a primary endpoint to test novel agents in the early setting. RCB is widely considered a prognostic factor and is frequently used as a surrogate endpoint for long-term outcomes, particularly in this BC subgroup. Although it is clear from recent meta-analyses that RCB is a better endpoint than pCR, the identification of the molecular characteristics that explain why some tumours do not follow the predicted outcomes (recurrences after excellent responses or long-lasting EFS after residual disease) continues to be a challenge. Robust evidence supports the association between RCB score and long-term outcome in patients twho have received NACT (79). Conversely, evidence for the predictive value of RCB in the context of targeted therapy is limited and requires further investigation. Multiple other methodologies to aid the identification of patients with higher disease relapse risk are currently being explored. The post neoadjuvant and adjuvant settings are an excellent opportunity to evaluate the contribution of dynamic biomarkers (e.g., RCB, TILs) to enable an accurate selection of patients who may benefit from escalating treatment strategies. Pre- and post-treatment assessment of ctDNA and TME plus integration of traditional transcriptomic and genomic signatures or classifications are some of the more promising approaches. Alternatively, innovative adaptive trial designs that enable early response assessment and facilitate an early change in management could minimise overtreatment and appropriately de-escalate or escalate therapy when appropriate.

Several molecular predictors of response that incorporate various ‘omic’ data to aid clinical decisions have been developed. Limited clinical impact has been derived due to lack of reproducibility, lengthy timeline of results, and expense. The real-time delivery of genomic and transcriptomic results will facilitate the implementation of adaptive trial designs and permit the investigation of novel and existing biomarkers. There are multiple pan-cancer studies assessing the implementation of genomics and transcriptomics into clinical care, for example, the UK 100,000 Genomes Project (217), the Dutch national Centre for Personalised Cancer Treatment (CPCT) study (218), and the Personalised Onco-Genomics (POG) Programme (219). The Personalised Breast Cancer Programme (PBCP) (220) is a tumour-specific precision medicine project that implements whole-genome sequencing data into the real-time treatment of early and advanced breast cancer patients. This programme ensures the delivery of high-quality annotated genomic data to patients and clinicians while promoting hypothesis testing and tumour-specific analysis. These large-scale sequencing studies will add considerably to our understanding and enable better optimisation of trial design, response prediction, and biomarker discovery. These efforts, combined with the promising potential of novel agents and treatment combinations, provide us with the exciting prospect of a tailored treatment pathway for each patient diagnosed with early-stage TNBC.

The ultimate aim is that every patient diagnosed with early-stage TNBC has a bespoke treatment pathway developed that fits their TNBC. The individualised use of preclinical models such as patient-derived organoids or xenografts (221), and the implementation of advanced radiodiagnostic techniques (222) are pivotal to achieving this goal. This type of integrated approach requires open and clear communication and collaboration between basic scientists, clinicians, and other scientific disciplines, for example, bioinformatics, mathematics, and physics, which will maximise the chance of success and ultimately enhance patient benefit.

In conclusion, advances in tumour characterisation, real-time biomarker/genomic testing, trial design, and drug development provide the foundation for an era of precision treatment in early TNBC. The development of complex strategies that integrate multi-modal data to derive individualised care plans, should consider the holistic needs of each patient to achieve a truly personalised approach.

## Author Contributions

KP, LD, RL, and JA each contributed to the design, literature review, writing, and editing of the manuscript. All authors listed have made a substantial, direct, and intellectual contribution to the work and approved it for publication.

## Funding

We would like to thank and acknowledge our funders, the Cancer Research UK (CRUK) (CRUK Cambridge Centre Grant Funding: RDZD141, RDZD192); Addenbrookes Charitable Trust (98100) and the Mark Foundation for Cancer Research (RDZD/174). This research was also supported by the NIHR Cambridge Biomedical Research Centre (BRC-1215-20014). The views expressed are those of the authors and not necessarily those of the NIHR or the Department of Health and Social Care.

## Acknowledgments

The authors want to thank the CRUK Precision Breast Cancer Institute, Cambridge and the Cambridge Cancer Trials Centre Womens’ Team for their support for our work in Triple Negative Breast Cancer. We also acknowledge the CRUK Cambridge Centre for its core support. We would like to thank the patients, families and carers who inspire us to work on TNBC.

## Conflict of Interest

The authors declare that the research was conducted in the absence of any commercial or financial relationships that could be construed as a potential conflict of interest.

## Publisher’s Note

All claims expressed in this article are solely those of the authors and do not necessarily represent those of their affiliated organizations, or those of the publisher, the editors and the reviewers. Any product that may be evaluated in this article, or claim that may be made by its manufacturer, is not guaranteed or endorsed by the publisher.

## Abbreviations

AR, Androgen receptor; BC, Breast cancer; BL1, Basal-like 1; BL2, Basal-like 2; BLIA, Basal-like immune activated; BLIS, Basal-like immunosuppressed; ctDNA, Circulating tumour DNA; DDR, DNA damage response; DFS, Disease free survival; DSBs, Double strand breaks; EBCTCG, Early Breast Cancer Trailist’s Collaborative Group; EFS, Event free survival; EMT, Epithelial-mesenchymal transition; ER, Oestrogen receptor; g*BRCA*, Germline *BRCA;* GE, Gene expression; HR, Homologous recombination; HRD, Homologous recombination deficiency; ICI, Immune checkpoint inhibitors; iDFS, Invasive disease free survival; IntClust, Integrative Cluster; iTILs, Intratumoural TILs; LAR, Luminal androgen receptor; M, Mesenchymal -Lehmann subtype; mAbs, Monoclonal antibodies; mTNBC, Metastatic triple negative breast cancer; NACT, Neoadjuvant chemotherapy; OR, Overall response; OS, Overall survival; PARP, Poly ADP-ribose polymerase; PARPi, Poly ADP-ribose polymerase inhibitors; pCR, Pathological complete response; PD-1, Programmed cell death 1; PD-L1, Programmed cell death ligand-1; PFS, Progression free survival; PR, Progesterone receptor; RCB, Residual Cancer Burden; RFS, Relapse-free survival; ssDNA, Single strand DNA; sTILs, Stromal TILs; TILs, Tumour infiltrating lymphocytes; TMB, Tumour mutational burden; TNBC, Triple negative breast cancer.

## Glossary

**Table:**

AR	Androgen receptor
BC	Breast cancer
BL1	Basal-like 1
BL2	Basal-like 2
BLIA	Basal-like immune activated
BLIS	Basal-like immunosuppressed
ctDNA	Circulating tumour DNA
DDR	DNA damage response
DFS	Disease free survival
DSBs	Double strand breaks
EBCTCG	Early Breast Cancer Trailist’s Collaborative Group
EFS	Event free survival
EMT	Epithelial-mesenchymal transition
ER	Oestrogen receptor
gBRCA	Germline BRCA
GE	Gene expression
HR	Homologous recombination
HRD	Homologous recombination deficiency
ICI	Immune checkpoint inhibitors
iDFS	Invasive disease free survival
IntClust	Integrative Cluster
iTILs	Intratumoural TILs
LAR	Luminal androgen receptor
M	Mesenchymal-Lehmann subtype
mAbs	Monoclonal antibodies
mTNBC	Metastatic triple negative breast cancer
NACT	Neoadjuvant chemotherapy
OR	Overall response
OS	Overall survival
PARP	Poly ADP-ribose polymerase
PARPi	Poly ADPribose polymerase inhibitors
pCR	Pathological complete response
PD-1	Programmed cell death 1
PD-L1	Programmed cell death ligand-1
PFS	Progression free survival
PR	Progesterone receptor
RCB	Residual Cancer Burden
RFS	Relapse-free survival
ssDNA	Single strand DNA
sTILs	Stromal TILs
TILs	Tumour infiltrating lymphocytes
TMB	Tumour mutational burden
TNBC	Triple negative breast cancer.
